# Epigenetic Mechanisms in Neurodegenerative Diseases: From Genetic Regulation to Therapeutic Potential

**DOI:** 10.3390/ijms27209322

**Published:** 2026-10-20

**Authors:** Xiaoman Lv, Mincong Huang, Wenjuan Long, Xinyao Li, Zhongyang Hu, Yujun Feng, Jiarong Du, Qiongrong Long, Yanxin Chen, Mingqin Shi, Dongdong Qin

**Affiliations:** 1 College of Pharmaceutical Science, Dali University, Dali 671003, China; lxm.cc@foxmail.com; 2 College of Chinese Materia Medica, Yunnan University of Chinese Medicine, Kunming 650500, China; hmc021228@foxmail.com; 3 Key Laboratory of Traditional Chinese Medicine for Prevention and Treatment of Neuropsychiatric Diseases, Yunnan University of Chinese Medicine, Kunming 650500, China; likq20201029@163.com (W.L.); xh18286279638@163.com (Z.H.);; 4 The First Clinical Medical College, Yunnan University of Chinese Medicine, Kunming 650500, China; lxy9081720@163.com

**Keywords:** neurodegenerative diseases, epigenetics, DNA methylation, histone modifications, non-coding RNAs, RNA methylation

## Abstract

Neurodegenerative diseases, including Alzheimer’s disease (AD), Parkinson’s disease (PD), and amyotrophic lateral sclerosis (ALS), are multifactorial disorders in which genetic susceptibility alone cannot account for sporadic onset, clinical diversity, or age-dependent progression. Epigenetic mechanisms—DNA methylation, histone modifications, non-coding RNAs, and m6A RNA methylation—bridge environmental and intracellular cues to stable gene expression changes. Emerging evidence associates epigenetic dysregulation with amyloid-β/tau pathology in AD, α-synuclein pathology in PD, and TDP-43/C9orf72-associated toxicity in ALS, while functional studies suggest that selected epigenetic alterations may contribute to these processes. In this narrative review, we distinguish associative, functional, and mechanistic evidence using claim-specific criteria based on experimental perturbation, direct molecular assessment, and pathway-dependence or rescue testing, while considering the study system. We further evaluate the translational promise of epigenetic biomarkers in liquid biopsies, while emphasizing the major barriers that separate bench findings from bedside application, including poor target specificity, limited blood–brain barrier penetration, and the absence of biomarker-guided patient stratification. By integrating pathological mechanisms, biomarker development, and therapeutic strategies, this review argues that future epigenetic research in neurodegeneration must prioritize causal validation, CNS-penetrant drug development, and prospective trials integrating epigenetic profiling to achieve meaningful clinical impact.

## 1. Introduction

### 1.1. Background and Rationale

Neurodegenerative diseases are a heterogeneous group of disorders characterized by the progressive loss of neuronal structure and function, resulting in cognitive decline, motor impairment, and reduced independence. Alzheimer’s disease (AD), Parkinson’s disease (PD), and amyotrophic lateral sclerosis (ALS) are among the most common and representative forms and impose a growing burden on public health and long-term care systems as the global population ages [1]. Although their clinical and pathological features differ, these diseases share several molecular processes, including abnormal protein aggregation, chronic neuroinflammation, mitochondrial dysfunction, oxidative stress, and glial activation. AD is primarily characterized by amyloid-beta (Aβ) and tau pathology. PD is accompanied by alpha-synuclein aggregation and dopaminergic neuron loss. ALS is characterized by TAR DNA-binding protein 43 (TDP-43) pathology and motor neuron degeneration [2,3].

Genetic studies have identified important disease-causing or risk-associated variants, including APP, PSEN1, and PSEN2 in familial AD; SNCA and LRRK2 in PD; and C9orf72 and SOD1 in ALS [4,5,6]. However, most patients present with sporadic or late-onset disease, and individuals with similar genetic backgrounds can differ substantially in age at onset, clinical manifestations, and rates of progression. The explanatory power of current genetic findings is further constrained by the underrepresentation of non-European populations and by the uncertain functional consequences of many risk variants located in non-coding regions [7]. Thus, genetic susceptibility alone does not fully account for disease heterogeneity or for the effects of aging, environmental exposure, lifestyle, and cellular stress.

Epigenetic regulation offers a framework for understanding how these factors influence disease-related gene expression without altering the underlying DNA sequence. DNA methylation, histone modifications, chromatin remodeling, non-coding RNAs, and RNA methylation can respond dynamically to aging, environmental stimuli, metabolic states, and neuronal activity [8,9]. In neurodegenerative diseases, disruption of these regulatory layers has been associated with pathological protein accumulation, synaptic dysfunction, neuroinflammation, and disease progression. Because some epigenetic alterations are measurable and potentially reversible, they may also provide biomarkers for diagnosis and patient stratification as well as targets for therapeutic intervention [10,11].

Genetic findings provide an essential framework for understanding neurodegeneration but do not fully explain environmental responses, age-dependent progression, or clinical heterogeneity. Epigenetic and epitranscriptomic regulation may help connect these factors with disease-related gene regulation. However, current findings vary in causal support, cellular context, and clinical maturity.

This review focuses on DNA methylation, histone modifications, and chromatin accessibility, together with related non-coding RNA and epitranscriptomic regulation. miRNA-mediated regulation is predominantly post-transcriptional, and m6A is an RNA modification, neither of which is uniformly equivalent to chromatin-based epigenetic regulation. They are included because of their interactions with disease-associated regulatory networks and are evaluated according to their specific mechanisms. Chromatin remodelers, histone variants, and three-dimensional genome organization are considered where directly relevant, but are not comprehensively reviewed.

### 1.2. Review Type and Literature Search Strategy

This article is a narrative review, not a systematic review or scoping review. It does not aim to provide an exhaustive, PRISMA-compliant synthesis of all available studies. Instead, it integrates representative evidence across AD, PD, and ALS to compare four regulatory layers, namely, DNA methylation, histone modifications, non-coding RNAs, and RNA methylation, to evaluate their biomarker and therapeutic potential.

We searched PubMed/MEDLINE, Web of Science, and Scopus from database inception to 31 August 2026. The search strategy combined three concept blocks: (i) disease terms: “neurodegenerative disease” OR “Alzheimer” OR “Parkinson” OR “amyotrophic lateral sclerosis” OR ALS; (ii) epigenetic/epitranscriptomic terms: “epigenetic” OR “DNA methylation” OR “5-hydroxymethylcytosine” OR “histone modification” OR “chromatin accessibility” OR “non-coding RNA” OR miRNA OR lncRNA OR circRNA OR “RNA methylation” OR m6A OR m5C OR m1A; and (iii) translation terms: “biomarker” OR diagnosis OR prognosis OR therapeutic OR “drug target” OR “clinical trial” OR “blood–brain barrier.” Reference lists of relevant reviews and key primary articles were also hand-searched.

We included English-language, peer-reviewed original research, clinical trials, meta-analyses, and reviews relevant to AD, PD, or ALS and to at least one epigenetic/epitranscriptomic mechanism, biomarker, or therapeutic strategy. We excluded non-English articles, preprints, and conference abstracts, except for one early-phase clinical report of an m6A-targeted agent (STC-15) for which no full-length report was available. Studies unrelated to AD, PD, or ALS; studies without epigenetic/epitranscriptomic measurements; and descriptive reports without disease-relevant outcomes were excluded.

Titles and abstracts were screened by two authors (X.L. and M.H.), and full texts were assessed for eligibility. Uncertainties were resolved by discussion with the senior authors (M.S. and D.Q.). Because this is a narrative review, study selection was purposive rather than exhaustive. For each claim, evidence was graded as associative, functional, or mechanistic according to the criteria in Section 2. Human clinical/cohort and human tissue evidence was prioritized for disease association. Mechanistic claims required experimental perturbation, direct molecular assessment, and pathway-dependence or rescue testing, usually in model systems. Animal and cell findings are identified as such and are not equated with human causality or clinical efficacy. The search was last updated on 31 August 2026.

## 2. Epigenetic Mechanisms in Major Neurodegenerative Diseases

Epigenetic dysregulation may link genetic susceptibility, environmental exposure, and aging to neurodegenerative pathology. However, the strength of evidence and biological effects vary across study systems. In AD, PD, and ALS, abnormalities in DNA methylation, histone modification imbalances, dysregulated expression of non-coding RNAs, and alterations in RNA methylation have been associated with these diseases, with functional or mechanistic involvement demonstrated for selected pathways in experimental models by modulating transcriptional activity, chromatin conformation, and post-transcriptional RNA homeostasis. Although the aforementioned three categories of neurodegenerative diseases share common pathogenic mechanisms—including protein homeostasis disruption, neuroinflammation, mitochondrial dysfunction, and synaptic abnormalities—AD is characterized primarily by Aβ deposition and tau protein pathology; PD is closely associated with abnormal aggregation of α-synuclein and degeneration of dopaminergic neurons; and the hallmark pathology of ALS involves pathological alterations in TDP-43/fused in sarcoma (FUS), C9orf72 hexanucleotide repeat expansion, and motor neuron damage.

For this narrative review, evidence is classified at the level of the specific claim rather than the study or regulator as a whole. Associative evidence comprises observed relationships between a regulatory alteration and disease-related measures without experimental perturbation. Functional evidence requires manipulation of the relevant regulator or alteration and a resulting change in a disease-related phenotype but does not establish the specific molecular pathway responsible. Mechanistic evidence requires a combination of perturbation, direct assessment of the proposed molecular intermediate, and pathway-dependence or rescue experiments supporting the link to the phenotype. Changes in regulator expression, global modification levels, target binding, or responses to nonspecific inhibitors alone are insufficient for mechanistic classification. Study species, tissue or cell type, and experimental setting are reported separately. Where these requirements are not met or findings conflict, the claim retains the lower supported category and the uncertainty is stated. These categories describe support for a particular claim. They are not a formal risk-of-bias assessment and do not establish causality or therapeutic efficacy in human disease.

### 2.1. Alzheimer’s Disease

#### 2.1.1. DNA Methylation

DNA methylation and hydroxymethylation alterations have been associated with AD-related pathology. DNA methyltransferases (DNMTs) catalyze the formation of 5-methylcytosine (5mC), whereas ten-eleven translocation (TET) enzymes oxidize 5mC to 5-hydroxymethylcytosine (5hmC), a modification enriched in the brain [9]. An epigenome-wide association study and meta-analysis of postmortem entorhinal cortex tissue identified 12 CpG sites significantly associated with AD case–control status or Braak stage. Six of these sites also showed significant correlations with mRNA expression. These findings link locus-specific DNA methylation differences to AD-related phenotypes and transcriptional variation but do not establish whether the methylation changes precede or cause disease progression [12].

Integrative methylomic, transcriptomic, and proteomic analyses have linked DNA methylation patterns to multiscale gene networks associated with AD. These results support network-level associations rather than demonstrating that methylation changes directly reshape these networks [13]. In addition to 5mC, genome-wide profiling of postmortem human brain tissue has identified differentially hydroxymethylated regions associated with amyloid-β and tau pathology. These findings implicate locus-specific 5hmC alterations in AD-associated molecular changes, without establishing a uniform global change or a causal role in disease onset [14]. Brain-derived 5hmC epigenetic scores have also been associated with AD neuropathology and cognitive decline. However, these scores were developed from postmortem brain samples and should not be presented as established diagnostic or prognostic tests for living patients [15].

Experimental evidence provides a distinct perspective on TET2-related regulation. In an AD mouse model, TET2-mutant myeloid cells showed increased CNS infiltration and enhanced phagocytic activity, accompanied by attenuation of AD-related pathology. These findings support a protective effect within the experimental setting but do not demonstrate that enhancing DNA demethylation is beneficial or establish therapeutic efficacy in patients [16]. Separately, a human observational study reported associations between peripheral-blood DNA methylation age measures and subsequent progression to cognitive impairment or AD [17]. Such findings do not establish that peripheral methylation profiles directly represent CNS epigenetic alterations. Overall, DNA methylation and hydroxymethylation findings in AD should be interpreted according to the tissue, cell type, genomic locus, and study design.

#### 2.1.2. Aberrant Histone Modifications

Altered histone modifications have been associated with Aβ deposition, tau pathology, synaptic injury, and neuroinflammation in AD. These alterations may influence disease-related transcription through changes in chromatin regulation. Histone acetylation, particularly H3K27ac, has received considerable attention. Cell-type-resolved analyses indicate that H3K27ac alterations differ among neurons, oligodendrocytes, and microglia. The enrichment of late-onset AD risk loci in microglia-specific regulatory regions further suggests a connection between genetic susceptibility and cell-type-specific gene regulation [18].

In neurons, H3K27ac is also closely related to the regulation of Aβ metabolism. Among these, studies of AD cell models show that a reduction in CREB-binding protein/p300 (CBP/p300) activity can reduce the level of H3K27ac and weaken some protective transcriptional processes that inhibit the generation of Aβ. Therefore, this change may promote the production of toxic Aβ substances. These findings show that changes in histone acetylation not only lead to changes in chromatin state but may also impair the protective responses of neurons that participate in Aβ pathology [19]. In AD research, in addition to the classic acetylation markers, several newer histone modification mechanisms have attracted more attention. For example, enhanced glycolysis in AD-associated microglia can increase histone H4 lysine 12 lactylation (H4K12la) and activate metabolic and inflammatory genes, including pyruvate kinase M2 (PKM2). This process may further form a glycolysis–H4K12la–PKM2 positive feedback loop, helping to sustain microglial inflammation and potentially aggravating Aβ pathology [20].

Abnormal histone methylation may also interfere with transcriptional regulation in AD. Recent evidence suggests that plant homeodomain finger protein 2 (PHF2), a histone demethylase, is upregulated in postmortem brain tissue from patients with AD, in AD patient-derived induced pluripotent stem cell (iPSC) neurons, and in five familial mutation Alzheimer’s disease (5xFAD) mice. Functionally, PHF2 can regulate inflammation-related genes, while PHF2 knockdown reduces inflammatory responses and partly improves synaptic function and cognitive behaviors. These findings indicate that disrupted histone methylation and demethylation may provide an epigenetic link between neuroinflammation and cognitive impairment [21]. In addition to acetylation and methylation, recent reviews have also emphasized that newer histone post-translational modifications, such as lactylation and crotonylation, may be involved in metabolic disturbance, inflammatory activation, and neuronal functional decline in AD [22].

Taken together, histone modification abnormalities in AD should not be viewed as isolated changes at individual modification sites. They are more likely to represent a broader process of epigenetic remodeling involving multiple histone marks. These changes may affect Aβ/tau pathology and synaptic function, while also interacting with impaired energy metabolism, glial reactivity, and neuroinflammation. Through these interconnected processes, abnormal histone modifications may eventually contribute to neuronal dysfunction and cognitive decline.

#### 2.1.3. Non-Coding RNA Abnormalities

Dysregulated non-coding RNAs have been implicated in AD-related processes, including Aβ metabolism, tau pathology, synaptic plasticity, neuroinflammation, and proteostasis. Among these RNAs, miRNAs have been extensively studied. miR-132 is consistently reported to be downregulated in AD brain tissue, and its restoration improves hippocampal neurogenesis and memory deficits in AD mouse models [23]. miR-132 also participates in the regulation of microglial states, suggesting that its effects extend beyond neurons to neuroimmune responses [24].

Long non-coding RNAs (lncRNAs) also participate in AD-related transcription disorders. Recent studies have found changes in m6A modification and expression patterns in promoter antisense RNAs and other non-coding transcripts derived from AD brain tissue. These changes may interfere with three-dimensional genome organisation and neuronal gene expression, leading to disease-related transcription imbalances [25]. In addition, FAM151B divergent transcripts (FAM151B-DT) are downregulated in brain regions that show tau pathology, including brain tissue in areas affected by AD. Reducing the expression of FAM151B-DT will affect autophagic flux and promote the accumulation of phosphorylated tau and easily aggregated proteins. This shows that lncRNAs participate in tau pathology by affecting protein clearance and autophagy-related processes [26].

Circular RNAs (circRNAs) are also dysregulated in the brain of patients with AD, and these changes may be closely related to the pathological pathway of irreversible disease progression [27]. For example, circular RNA (circAPP) derived from amyloid precursor protein can regulate microglial polarization through the axis of miR-1906/chloride intracellular channel 1 (CLIC1), thus promoting inflammatory response and cognitive impairment. In addition, reducing the level of circAPP or targeting its downstream pathway has been proven to alleviate the AD-like pathology in APP/PS1 mice [28]. In general, non-coding RNA abnormalities in AD should not be regarded as independent changes in a single molecule but as a broader destruction of the regulatory network of miRNAs, lncRNAs and circRNAs. These changes may interact with Aβ/tau pathology, synaptic damage, impaired autophagy and glial-driven inflammation and eventually lead to progressive cognitive decline.

#### 2.1.4. Aberrant RNA Methylation

In AD, RNA methylation abnormalities are mainly manifested as m6A modification disorders. m6A can be involved in regulating RNA stability, splicing, transport, translation efficiency and non-coding RNA functions. Therefore, when this regulatory process is disordered, it may participate in amyloid-β (Aβ) deposition, tau pathology, synaptic damage and AD-related pathological processes such as inflammation. Methyltransferases, such as methyltransferase-like protein 3 (METTL3) and methyltransferase-like protein 14 (METTL14), are mainly responsible for adding m6A marks to the target RNA; correspondingly, demethylases, such as fat mass and obesity-associated protein (FTO) and AlkB homolog 5 (ALKBH5), can remove these markers. The reader proteins of the YTH domain family (YTHDF) and YTH domain-containing (YTHDC) families can identify m6A-modified RNA and further affect its downstream fate. Studies have shown that neuronal m6A levels and METTL3 expression in the brain tissue of AD patients may be reduced. In addition, METTL3 knockdown can induce abnormal cell cycle re-entry, oxidative stress, synaptic loss and neuronal death, which shows that the METTL3–m6A axis plays an important role in maintaining neuronal homeostasis and cognitive function [29]. The expression of m6A writers, erasers, and readers should be distinguished from RNA methylation itself. Changes in regulator abundance do not establish corresponding changes in catalytic activity, global m6A levels, or modification of individual transcripts. Reader expression may alter the fate of modified RNAs without changing methylation abundance. Direct measurements of RNA modification, together with transcript-specific and functional analyses, are therefore required to interpret these findings.

However, m6A dysregulation in AD should not be understood simply as a global increase or decrease in RNA methylation. It is more likely to reflect a context-dependent disturbance of RNA methylation homeostasis, and this disturbance may vary across different cell types, disease stages, and target RNAs. Recent m6A profiling of the human brain has shown that m6A alterations in the frontal cortex of patients with AD occur not only in mRNAs but also in non-coding transcripts, such as promoter-associated antisense RNAs. Among them, microtubule-associated protein tau-associated promoter antisense RNA (MAPT-paRNA) is regulated by m6A and may affect neuronal and synapse-related gene expression through three-dimensional genome interactions. In this way, it could contribute to impaired neuronal survival and AD-related transcriptional imbalance [25]. In addition, m6A modification can influence synaptic function through circRNAs. In APP/PS1 mice, METTL3-dependent m6A modification stabilizes RIMS2-derived circular RNA (circRIMS2). This stabilized circRIMS2 then promotes glutamate ionotropic receptor NMDA type subunit 2B (GluN2B) ubiquitination and degradation through the miR-3968/ubiquitin-conjugating enzyme E2 K (UBE2K) axis, eventually leading to synaptic damage and memory impairment [30].

METTL3-mediated m6A modification also plays an important role in immune regulation and Aβ clearance. Loss of METTL3 in myeloid cells reduces m6A modification and translation of DNA methyltransferase 3 alpha (Dnmt3a) mRNA. At the same time, it enhances the migration of monocyte-derived macrophages into the brain and improves their ability to clear Aβ, thereby alleviating AD-like pathology and cognitive deficits [31]. By contrast, FTO downregulation can increase m6A modification of Notch receptor 1 (Notch1) mRNA and activate the Notch1–hairy and enhancer of split 1 (Hes1) inflammatory pathway. This finding suggests that impaired m6A demethylation may also contribute to AD progression by modulating immune responses [32]. These findings indicate that m6A regulation has context-dependent effects. METTL3 depletion can impair neuronal homeostasis, whereas its loss in monocyte-derived macrophages ameliorates pathology in the reported mouse model [29,31]. Therefore, neither increased nor decreased m6A should be interpreted as uniformly pathogenic or protective.

In addition to m6A, other types of RNA methylation modifications, including N1-methyladenosine (m1A) and 5-methylcytosine (m5C), have also gradually received attention from AD researchers in recent years. In AD models, the abnormal m1A modification of mitochondrial and cytoplasmic tRNA may affect tRNA stability and translation efficiency and further aggravate mitochondrial dysfunction [33]. At the same time, altered expression of the m5C-related methyltransferases NOP2/Sun RNA methyltransferase 6 (NSUN6) and NOP2/Sun RNA methyltransferase 7 (NSUN7), as well as the m5C reader Aly/REF export factor (ALYREF), was also observed in AD brain tissue. These results suggest that m5C may be involved in AD-related RNA metabolic abnormalities and have a certain relationship with the pathological progression of the disease, but its specific mechanism of action still needs to be further verified [34]. Compared with m6A, the evidence for m1A and m5C findings discussed here is limited. Altered expression of NSUN6, NSUN7, or ALYREF should not be equated with demonstrated changes in RNA m5C [34]. These observations should therefore be distinguished from direct modification measurements and functional perturbation studies, and their contribution to AD remains to be established.

In general, abnormal RNA methylation in AD cannot be simply understood as an independent surface transcriptome event. On the contrary, it is more likely to be part of the disorder of the overall RNA regulatory network and may further participate in the disease progression of AD by affecting neuronal survival, synaptic plasticity, Aβ/tau pathology, non-coding RNA regulation and glial cell inflammatory response.

**Figure 1:**
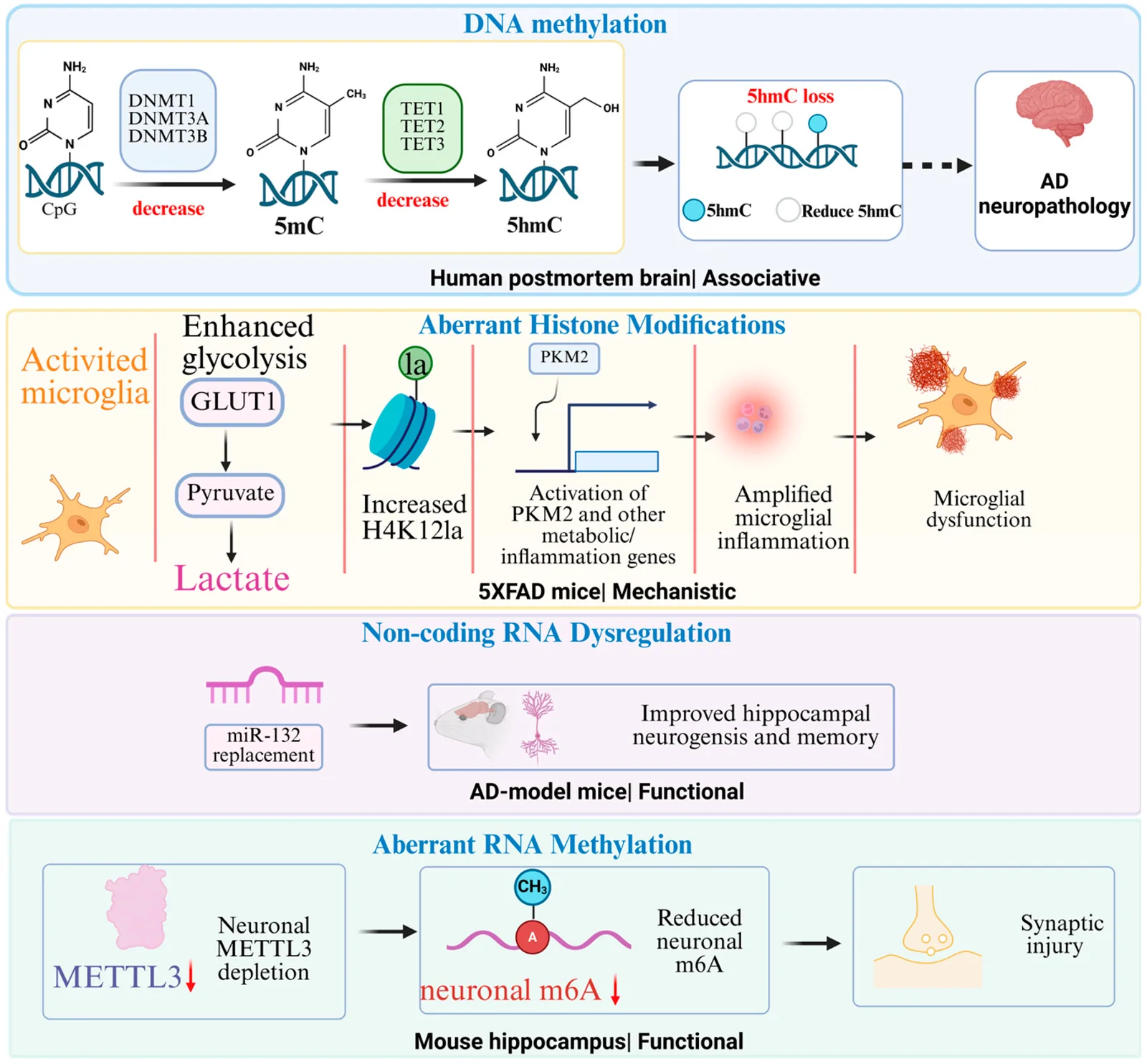
Selected regulatory findings in AD. Panels show locus-specific DNA methylation/hydroxymethylation associations, the microglial H4K12 lactylation–PKM2 pathway, miR-132 replacement in AD-model mice, and neuronal METTL3 depletion in the mouse hippocampus. The DNA panel does not imply uniform global 5mC or 5hmC loss. H4K12la, histone H4 lysine 12 lactylation; PKM2, pyruvate kinase M2; m6A, N^6^-methyladenosine. Labels distinguish associative observations, functional perturbation evidence, and mechanistic pathway evidence in the indicated study systems. Dashed connections denote association, solid arrows denote experimentally supported effects and the red downward arrow indicates downregulation. Model findings do not establish causality or therapeutic efficacy in patients. (Created in BioRender. Xing, Li. (2026). https://BioRender.com/2fwkvyx, accessed on 13 October 2026).

Epigenetic regulation plays a pivotal role in the pathogenesis of AD, influencing disease progression by modulating gene expression levels and cellular response processes. Abnormal alterations in epigenetic modifications—including DNA methylation, histone modifications, non-coding RNAs, and RNA methylation—collectively mediate the development of Aβ/tau pathology, synaptic dysfunction, neuroinflammatory activation, and neuronal damage. The key epigenetic mechanisms involved in AD progression are summarized in Figure 1.

### 2.2. Parkinson’s Disease

#### 2.2.1. DNA Methylation

In PD, abnormal DNA methylation is related to the onset and progression of the disease. It may affect several PD-related processes, including α-synuclein expression, oxidative stress, lysosomal function and neuroinflammatory response. Among the DNA methylation-related genes studied in PD, SNCA has received widespread attention. Reduced DNA methylation at SNCA intron 1 has been observed in the frontal cortex of individuals with GBA1-associated PD [35]. This finding provides associative evidence of altered epigenetic regulation at the SNCA locus. However, the study did not directly establish that this methylation change increases SNCA expression or causes α-synuclein accumulation.

In addition to SNCA, a study has found gender-specific DNA methylation changes in the neurons of patients with Parkinson’s disease. These changes involve multiple Parkinson’s disease-related genes, including Parkinson’s disease-related deglycase (PARK7), solute carrier family member 6 (SLC17A6), protein tyrosine phosphatase receptor type N2 (PTPRN2) and nuclear receptor subfamily 4 group A member 2 (NR4A2). Since most of the above genes are involved in oxidative stress regulation, neurotransmitter release and dopaminergic neuronal identity maintenance, it is currently speculated that abnormal methylation can disrupt neuronal homeostasis through multiple pathways [36]. In addition to 5-methylcytosine (5mC), abnormally altered 5-hydroxymethylcytosine (5hmC) has also become an important direction for epigenetic research on Parkinson’s disease. A study found a large number of differentially hydroxymethylated regions were detected in the substantia nigra of patients with Parkinson’s disease, which were mainly concentrated in genes related to nerve generation, axon function and signal transduction. These findings identify PD-associated hydroxymethylation differences in the substantia nigra but do not establish whether these alterations precede or contribute to neuronal degeneration [37].

Recent studies have further revealed the abnormal 5mC/5hmC conversion kinetics in PD neurons. These changes involve genes related to Ras-related protein Rab-32 (RAB32), ArfGAP with GTPase domain, ankyrin repetition and PH domain 1 (AGAP1), LRRK2-mediated endosomal trafficking and neuroinflammatory networks. This phenomenon shows that DNA methylation and hydroxymethylation cannot be regarded as independent epigenetic changes. On the contrary, they may work together in the epigenetic regulation of PD pathology [38]. In addition, studies using peripheral immune cells and blood-based methylomes have identified DNA methylation changes associated with PD. Peripheral methylation differences have been associated with PD, but their correspondence with CNS alterations and their clinical utility remain to be established [39,40].

In general, DNA methylation abnormalities in PD are unlikely to be isolated changes in a single locus. On the contrary, these anomalies are more likely to reflect a dynamic process of epigenetic remodeling shaped by genetic background, brain region, cell type and environmental factors. In this process, DNA methylation with abnormal changes may promote the progression of PD by affecting the accumulation of α-synuclein, oxidative stress, endolysosomal dysfunction and neuroinflammatory response.

#### 2.2.2. Aberrant Histone Modifications

In PD, abnormal histone modifications are closely associated with α-synuclein aggregation, mitochondrial injury, synaptic dysfunction, and neuroinflammation. By altering chromatin accessibility and the transcriptional activity of key disease-related genes, these changes may influence the course of PD progression. Among the different types of histone modifications, histone acetylation has received particular attention. Previous studies have shown that histone H3 lysine 27 acetylation (H3K27ac) is markedly increased in the brains of patients with PD. H3K27ac is also enriched at several PD-associated gene loci, including SNCA, PARK7, parkin RBR E3 ubiquitin protein ligase (PRKN), and MAPT. Altered H3K27ac patterns at PD-associated loci suggest changes in regulatory activity, but these observations alone do not establish their functional consequences or causal contribution to PD [41].

Mitochondrial dysfunction may also drive abnormal histone acetylation in PD. Under complex I impairment or mitochondrial stress, dopaminergic neurons show increased H3K27ac levels, along with disrupted enhancer activity and altered downstream gene expression. This points to a possible pathological connection between mitochondrial injury, H3K27ac dysregulation, and transcriptional imbalance [42]. Impaired energy metabolism can further influence histone acetylation at autophagy-related genes. For example, reduced aconitase 2 (ACO2) function suppresses histone H3 lysine 9 (H3K9) and histone H4 lysine 5 (H4K5) acetylation, leading to decreased transcription of autophagy genes such as microtubule-associated protein 1 light chain 3 (LC3) and autophagy-related 5 (Atg). As a result, α-synuclein clearance may be weakened, and dopaminergic neurons may become more vulnerable to degeneration [43].

In addition to acetylation, aberrant histone methylation also contributes to PD progression. Increased histone H3 lysine 4 trimethylation (H3K4me3), which is generally considered an active histone mark, at the SNCA promoter may promote α-synuclein expression. Conversely, targeted reduction in H3K4me3 at this locus using a clustered regularly interspaced short palindromic repeats/deactivated Cas9 (CRISPR/dCas9)-based approach can lower α-synuclein levels. This suggests that locus-specific modulation of histone modifications may have therapeutic potential [44]. In parallel, α-synuclein pathology can induce euchromatic histone lysine methyltransferase 1/2 (EHMT1/2) upregulation and increase histone H3 lysine 9 dimethylation (H3K9me2). This change may repress synapse-related genes, such as synaptosomal-associated protein 25 (SNAP25), postsynaptic density protein 95 (PSD95), Synapsin 1, and vesicular glutamate transporter 1 (vGLUT1). As a result, synaptic injury and motor dysfunction may become more severe, while EHMT1/2 inhibition in disease models can partly reduce these deficits [45].

In addition, several emerging histone modifications have begun to attract attention in PD mechanistic studies. Enhanced glycolysis in microglia can cause lactate accumulation and promote p300/CBP-mediated H3K9 lactylation. Increased H3K9 lactylation may further activate inflammatory genes, such as solute carrier family 7 member 11 (SLC7A11), thereby promoting microglial activation and dopaminergic neuronal injury [46]. Taken together, histone modification abnormalities in PD are unlikely to represent isolated changes at individual modification sites. Instead, they appear to be closely connected with α-synuclein pathology, mitochondrial metabolic dysfunction, impaired autophagy, synaptic loss, and neuroinflammation. Through the combined effects of these processes, abnormal histone modifications may contribute to dopaminergic neurodegeneration.

#### 2.2.3. Non-Coding RNA Abnormalities

In PD, dysregulated non-coding RNAs are closely involved in disease progression. These RNAs may influence several PD-related processes, including α-synuclein expression, mitochondrial homeostasis, autophagic clearance, oxidative stress, and neuroinflammation. Among miRNAs, miR-7 has been widely studied because of its regulatory effect on α-synuclein. Restoring miR-7 expression can partly reduce α-synuclein preformed fibril (α-syn PFF)-induced pathological spreading and neuroinflammatory responses, which may help protect dopaminergic neurons from injury [47]. In addition, miR-218-5p is downregulated in PD models, and its overexpression can attenuate microglial inflammation by inhibiting DEAD-box helicase 41 (Ddx41)/type I interferon signaling, ultimately exerting a protective effect on nigral dopaminergic neurons [48]. Dysregulated autophagy-related miRNAs may also affect α-synuclein clearance. For example, increased miR-214-3p can suppress autophagy, thereby further promoting PD-related pathology [49].

lncRNAs often act as competing endogenous RNAs and regulate miRNA–mRNA axes, thereby influencing the expression of PD-related genes. Recent studies have shown that HOXA transcript antisense RNA myeloid-specific 1 (HOTAIRM1) can modulate α-synuclein expression by sponging miR-223-3p. Moreover, regulation of the HOTAIRM1/miR-223-3p/α-syn axis can partly alleviate dopaminergic neuronal injury associated with α-syn A53T [50]. circRNAs are also increasingly recognized as important post-transcriptional regulators in PD. Many PD-related circRNAs are enriched in neurons and may function as miRNA sponges, thereby affecting synaptic function and neurotoxicity. Cell type-specific circRNA profiling of the human brain has further revealed circRNA alterations associated with PD risk genes in dopaminergic neurons. This suggests that circRNAs may participate in pathological post-transcriptional regulation within vulnerable neuronal populations [51].

Further mechanistic studies have shown that pantothenate kinase 1-derived circular RNA (circ-Pank1) is upregulated in PD models and can increase α-synuclein expression by sponging miR-7a-5p. This process may aggravate motor impairment and dopaminergic neurodegeneration [52]. By contrast, synaptic vesicle glycoprotein 2B-derived circular RNA (circSV2b) appears to exert a protective effect by reducing oxidative damage through the miR-5107-5p/forkhead box K1 (Foxk1)/AKT serine/threonine kinase 1 (Akt1) axis. These findings indicate that circRNAs do not act in a uniform manner in PD; depending on their targets and regulatory pathways, they may have either pathogenic or protective roles [53]. Taken together, non-coding RNA abnormalities in PD are unlikely to represent isolated changes in individual molecules. Rather, they may reflect a broader disruption of post-transcriptional regulatory networks involving miRNAs, lncRNAs, and circRNAs. Through their effects on α-synuclein aggregation, impaired autophagy, oxidative stress, neuroinflammation, and dopaminergic neuronal death, these regulatory changes may jointly contribute to PD progression.

#### 2.2.4. Aberrant RNA Methylation

Abnormal RNA methylation is an important component of epitranscriptomic dysregulation in PD. Among the different RNA methylation marks, N6-methyladenosine (m6A) has been the most extensively studied. m6A marks are mainly installed by methyltransferases such as METTL3 and METTL14, removed by demethylases such as FTO and ALKBH5, and recognized by reader proteins from the YTHDF and insulin-like growth factor 2 mRNA-binding protein (IGF2BP) families. Together, these enzymes and reader proteins help determine the fate of target RNAs. Through this regulatory system, m6A modification can influence mRNA stability, splicing, and translation. Studies of brain tissue from Lewy body-related disorders have revealed abnormal changes in both the overall level and subcellular distribution of m6A modification, suggesting that disrupted RNA methylation may be involved in α-synuclein-related pathology [54].

In PD, reduced METTL14 expression weakens m6A modification of α-syn mRNA and impairs YTHDF2-mediated RNA degradation, which may promote abnormal α-synuclein accumulation [55]. In parallel, dysfunction of METTL3 or METTL14 can further disturb several neuronal survival-related processes, including mitochondrial transcription factor A (TFAM)-mediated mitochondrial biogenesis, ATPase sarcoplasmic/endoplasmic reticulum Ca^2+^ transporting 3 (ATP2A3)-related endoplasmic reticulum calcium homeostasis, and oxidative stress responses. These disruptions may ultimately exacerbate nigral dopaminergic neuronal injury [56,57]. Conversely, aberrant upregulation of the demethylase FTO can reduce m6A modification of specific target mRNAs and promote neuronal death and ferroptosis through pathways involving ataxia telangiectasia mutated (ATM) and BRCA1-associated protein 1 (BAP1)/tumor protein p53 (p53)/SLC7A11 [58,59]. Recent evidence also suggests that pathological α-synuclein can induce abnormal METTL3 activation in microglia, leading to increased expression of inflammatory cytokines. These findings indicate that RNA methylation abnormalities not only disrupt neuronal homeostasis but may also promote PD progression through glial cell-mediated neuroinflammatory responses [60].

**Figure 2:**
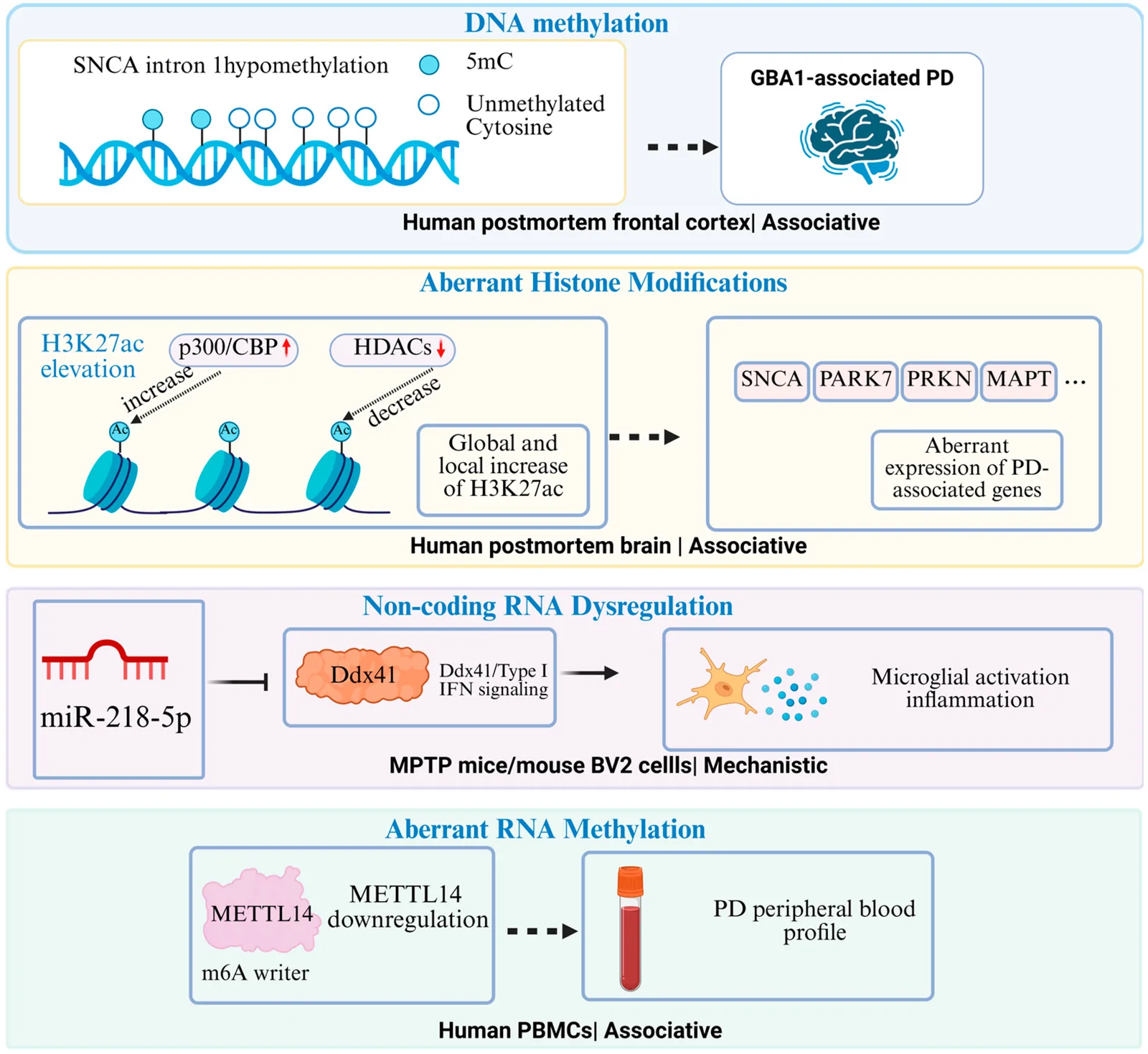
Selected regulatory findings in PD. Panels show SNCA intron 1 methylation in GBA1-associated PD, H3K27ac alterations in postmortem brain, miR-218-5p–Ddx41/type I interferon regulation in mouse models and BV2 cells, and reduced METTL14 and m6A in human peripheral blood mononuclear cells (PBMCs). Blood associations do not establish corresponding brain changes. H3K27ac, histone H3 lysine 27 acetylation; IFN, interferon; SNCA encodes α-synuclein. Labels distinguish associative observations, functional perturbation evidence, and mechanistic pathway evidence in the indicated study systems. Dashed connections denote association, and solid arrows denote experimentally supported effects. A red upward arrow indicates upregulation, and a red downward arrow indicates downregulation. Black T-bars denote inhibition. Model findings do not establish causality or therapeutic efficacy in patients. (Created in BioRender. Xing, Li (2026). https://BioRender.com/2fwkvyx, accessed on 13 October 2026).

Epigenetic regulatory mechanisms, by modulating gene expression and maintaining the homeostasis of the neuronal microenvironment, have become a critical regulatory level in the pathogenesis and progression of Parkinson’s disease (PD). Abnormal alterations in epigenetic modifications—including DNA methylation, histone modifications, non-coding RNAs, and RNA methylation—collectively contribute to the development of α-synuclein pathology, mitochondrial dysfunction, synaptic damage, neuroinflammatory activation, and degeneration of dopaminergic neurons. The major epigenetic mechanisms involved in PD progression are summarized in Figure 2.

### 2.3. Amyotrophic Lateral Sclerosis

#### 2.3.1. DNA Methylation

Aberrant DNA methylation is an important component of epigenetic dysregulation in ALS. It involves genome-wide changes in CpG methylation, abnormal promoter methylation of specific genes, disrupted mitochondrial DNA methylation, and accelerated epigenetic aging. Large-scale blood-based epigenome-wide association studies (EWASs) have identified several differentially methylated sites in patients with ALS. Genes linked to these sites are mainly enriched in pathways related to lipid metabolism, cholesterol biosynthesis, and immune and inflammatory responses. This suggests that DNA methylation may provide a link between environmental exposure, metabolic disturbance, and immune activation during ALS progression [61]. Further studies have also shown that DNA methylation changes in ALS are associated with disease progression rate and survival. The corresponding differentially methylated regions are mainly enriched in pathways related to synapses, axons, and neuronal function. Therefore, methylation abnormalities may aggravate motor neuron degeneration by affecting neuronal structural stability and axonal function [62].

At the mechanistic level, increased expression of DNA methyltransferase 1 (DNMT1) and DNA methyltransferase 3A (DNMT3A), together with elevated 5-methylcytosine (5mC) levels, has been observed in ALS models and human ALS tissues. These alterations may lead to abnormal promoter hypermethylation in motor neurons and skeletal muscle cells, further affecting mitochondrial function, cytoskeletal stability, proteostasis, and cell death-related pathways. In SOD1-G93A mice, treatment with DNMT inhibitors improves motor function and prolongs survival. This finding suggests that abnormal DNA methylation is not merely a disease-associated change but may also actively contribute to ALS pathogenesis [63]. In addition, reduced methylation of the mitochondrial DNA displacement loop (D-loop) region has been detected in peripheral blood from patients with ALS. This change is associated with altered mitochondrial DNA copy number, suggesting a possible link among mitochondrial DNA methylation imbalance, mitochondrial replication, impaired energy metabolism, and oxidative stress [64].

Notably, in C9orf72 repeat expansion-associated ALS/frontotemporal dementia (FTD), hypermethylation of the C9orf72 promoter or repeat region may carry a different implication. This alteration may have a protective effect by limiting toxic repeat-derived products. However, its interpretation as a compensatory response requires evidence that it arises in response to pathological stress and mitigates subsequent injury [65]. In recent years, accelerated DNA methylation age has also been linked to earlier disease onset, shorter survival, and occupational exposure in ALS. This further suggests that DNA methylation abnormalities may connect aging, environmental factors, and motor neuron vulnerability [66,67].

Taken together, abnormal DNA methylation in ALS may contribute to disease onset and progression by affecting inflammatory responses, lipid metabolism, mitochondrial homeostasis, axonal function, and cell death. However, the causal relationships and cell type-specific effects underlying these associations still require further investigation.

#### 2.3.2. Aberrant Histone Modifications

In the pathogenesis of ALS, abnormal histone modification may be involved in regulating disease onset and progression. These alterations can further affect many core pathological processes of ALS by regulating chromatin accessibility, motor neuron stress responses and disease-related genes. Dysregulated histone acetylation is one of the most critical epigenetic abnormalities in this disease. In the ALS model related to fused in sarcoma (FUS), the level of global histone acetylation was significantly reduced, accompanied by abnormal expression of metabolism-related genes. Histone deacetylase (HDAC) inhibitor intervention that can pass through the blood–brain barrier can partially restore the level of histone acetylation, correct metabolic abnormalities, and prolong the survival of model animals. HDAC inhibition restored histone acetylation and improved selected outcomes in FUS-overexpressing mice. These findings support functional effects in this model but do not establish that acetylation changes mediate all observed benefits or demonstrate efficacy in patients [68].

ALS-associated RNA-binding proteins, such as FUS and TDP-43, are not only involved in RNA metabolism but can also influence chromatin remodeling and transcriptional regulation. When these proteins lose their normal nuclear functions or abnormally aggregate in the cytoplasm, several regulatory processes may be disturbed, including enhancer–promoter interactions, RNA–DNA hybrid loop structure (R-loop) homeostasis, and chromatin architecture. As a result, cells may develop broader transcriptional imbalance, together with abnormal activation of transposable elements [69]. In C9orf72 repeat expansion-associated ALS/frontotemporal dementia (FTD), single-cell transcriptomic and epigenomic studies have also reported abnormal chromatin accessibility and altered H3K27ac in the motor and frontal cortices, accompanied by related changes in gene expression. These alterations appear to be particularly evident in excitatory neurons and astrocytes, suggesting that active histone acetylation marks may contribute to neuronal functional decline, proteostatic stress, and enhanced glial reactivity [70].

Beyond acetylation, abnormal histone methylation is also closely related to ALS. In the C9orf72 repeat expansion region, several repressive histone methylation marks are enriched, including histone H3 lysine 9 trimethylation (H3K9me3), histone H3 lysine 27 trimethylation (H3K27me3), histone H3 lysine 79 trimethylation (H3K79me3), and histone H4 lysine 20 trimethylation (H4K20me3). These marks may promote C9orf72 transcriptional silencing and haploinsufficiency, thereby affecting autophagy, endosomal trafficking, and neuroinflammatory regulation [71]. In addition, age-associated increases in suppressor of variegation 3–9 homolog 1 (SUV39H1)/Su(var)3–9 activity can enhance H3K9 methylation at the TARDBP/TDP-43 promoter. This change may reduce TDP-43 expression and induce motor dysfunction. Together, these findings suggest that age-dependent remodeling of histone methylation may provide an important epigenetic basis for increased ALS susceptibility [72].

Taken together, histone modification abnormalities in ALS are unlikely to arise from a single isolated modification event. Rather, they appear to be closely connected with FUS/TDP-43 proteostasis imbalance, C9orf72 repeat expansion, mitochondrial and metabolic dysfunction, DNA damage responses, and glial activation. Through the combined effects of these processes, abnormal histone modifications may contribute to motor neuron degeneration [73].

#### 2.3.3. Non-Coding RNA Abnormality

In ALS, dysregulated non-coding RNAs are involved in both disease onset and progression. These RNAs can influence several ALS-related processes, including RNA metabolism, neuromuscular junction homeostasis, glial responses, and the function of RNA-binding proteins. Among miRNAs, motor neuron-enriched miR-218 appears to be particularly vulnerable to dysregulation in ALS. Reduced miR-218 expression may disrupt gene regulatory networks within motor neurons, thereby affecting axonal growth, neuromuscular junction maintenance, and muscle innervation [74]. Meanwhile, reduced levels of muscle-derived extracellular vesicle miR-126a-5p may promote abnormal local synthesis and accumulation of TDP-43 in axons and neuromuscular junctions, which may further aggravate neuromuscular junction degeneration [75].

Among lncRNAs, nuclear paraspeckle assembly transcript 1 (NEAT1) has received considerable attention in ALS research. As a structural scaffold of paraspeckles, NEAT1 is closely involved in nuclear RNA homeostasis and stress-response regulation through its interactions with RNA-binding proteins such as TDP-43 and FUS. Previous studies have suggested that NEAT1-related genetic variants are associated with age at ALS onset. Meanwhile, loss of nuclear TDP-43 function can reduce NEAT1 expression and impair its contribution to protein homeostasis and cellular stress responses [76,77]. In addition, TDP-43 can regulate paraspeckle condensation through its interaction with nuclear paraspeckle assembly transcript 1 isoform 2 (NEAT1-2). This suggests that abnormalities in lncRNA–protein condensates may serve as an important link between RNA metabolic dysfunction and protein pathology in ALS [78].

At the circRNA level, C9orf72 repeat expansion can generate circular intronic RNA, which is exported from the nucleus and acts as an aberrant translation template for the production of dipeptide repeat proteins. Through this process, it may mediate both RNA toxicity and protein toxicity [79]. Meanwhile, circRNA expression in ALS models and patients shows tissue-dependent alterations. This suggests that circRNAs may further influence pathological responses in neurons and skeletal muscle, either by acting as miRNA sponges or by interacting with RNA-binding proteins [80]. Taken together, non-coding RNA abnormalities in ALS are unlikely to be isolated molecular changes. Instead, they may reflect a broader disruption of RNA regulatory networks involving miRNAs, lncRNAs, and circRNAs. These alterations are closely linked to TDP-43/FUS pathology, C9orf72 repeat RNA toxicity, neuromuscular junction disruption, and glial dysfunction. Through these interconnected processes, dysregulated non-coding RNAs may contribute to motor neuron degeneration.

#### 2.3.4. Aberrant RNA Methylation

In ALS, RNA methylation abnormalities are mainly reflected in disrupted m6A homeostasis. These changes may influence RNA stability, splicing, transport, and translation, and may therefore contribute to motor neuron degeneration. In C9orf72 repeat expansion-associated ALS/frontotemporal dementia (FTD), global reduction in m6A can impair YTH domain-containing protein 1 (YTHDC1)-mediated degradation of repeat-containing RNAs. As a result, repeat RNAs and dipeptide repeat proteins may accumulate, further increasing neurotoxicity [81]. In ALS associated with TDP-43 pathology, m6A modification can also affect the binding of TDP-43 to target RNAs, as well as the regulation of TDP-43 expression itself. In this context, dysregulation of the m6A reader YTHDF2 may further enhance TDP-43-related neurotoxicity [82].

In FUS mutation-associated ALS, increased m6A levels may promote the formation of aberrant stress granules, whereas reduced METTL3 activity decreases the accumulation of FUS-positive granules. This suggests that m6A may participate in ALS pathogenesis by regulating RNA–protein condensates and phase-separation dynamics [83]. At the level of neuronal homeostasis, METTL3- or METTL14-mediated m6A modification is also important for maintaining motor neuron function and neuromuscular junction integrity. Loss of Mettl3 or Mettl14 can lead to motor impairment, motor neuron loss, or ALS-like phenotypes, while FTO inhibition, which increases m6A levels, can partly alleviate neurodegeneration in disease models [84,85].

Beyond m6A, 5-methylcytosine (m5C) has also been increasingly linked to amyotrophic lateral sclerosis/frontotemporal dementia (ALS/FTD) pathology. NOP2/Sun RNA methyltransferase 1 (NSUN1)-mediated m5C RNA methylation may promote the cytoplasmic accumulation of TDP-43 and increase neurotoxicity. This finding suggests that m5C dysregulation may represent another pathway through which RNA modification imbalance contributes to TDP-43 protein pathology [86]. Taken together, RNA methylation abnormalities in ALS should not be viewed as a mere increase or decrease in a single RNA modification. Rather, they appear to reflect a broader epitranscriptomic imbalance associated with C9orf72 repeat RNA toxicity, TDP-43/FUS protein pathology, stress granule dynamics, and disrupted motor neuron homeostasis. The NSUN1 study provides experimental support for a connection between m5C regulation and TDP-43 toxicity [86]. However, this mechanism-specific evidence does not establish comparable maturity of the broader m5C and m6A evidence bases or validated biomarker and therapeutic applications.

Functional interpretation also requires transcript- and cell-specific information. A global m6A measurement may conceal different changes among individual RNAs or cell populations, while modification of different transcripts can produce distinct effects on RNA stability, translation, or processing. For example, neuronal METTL3 depletion and its loss in monocyte-derived macrophages produce different outcomes in the reported AD-related models [29,31]. Thus, neither regulator expression nor global m6A abundance alone identifies the affected RNA pathway or establishes whether the alteration is pathogenic, protective, or secondary.

**Figure 3:**
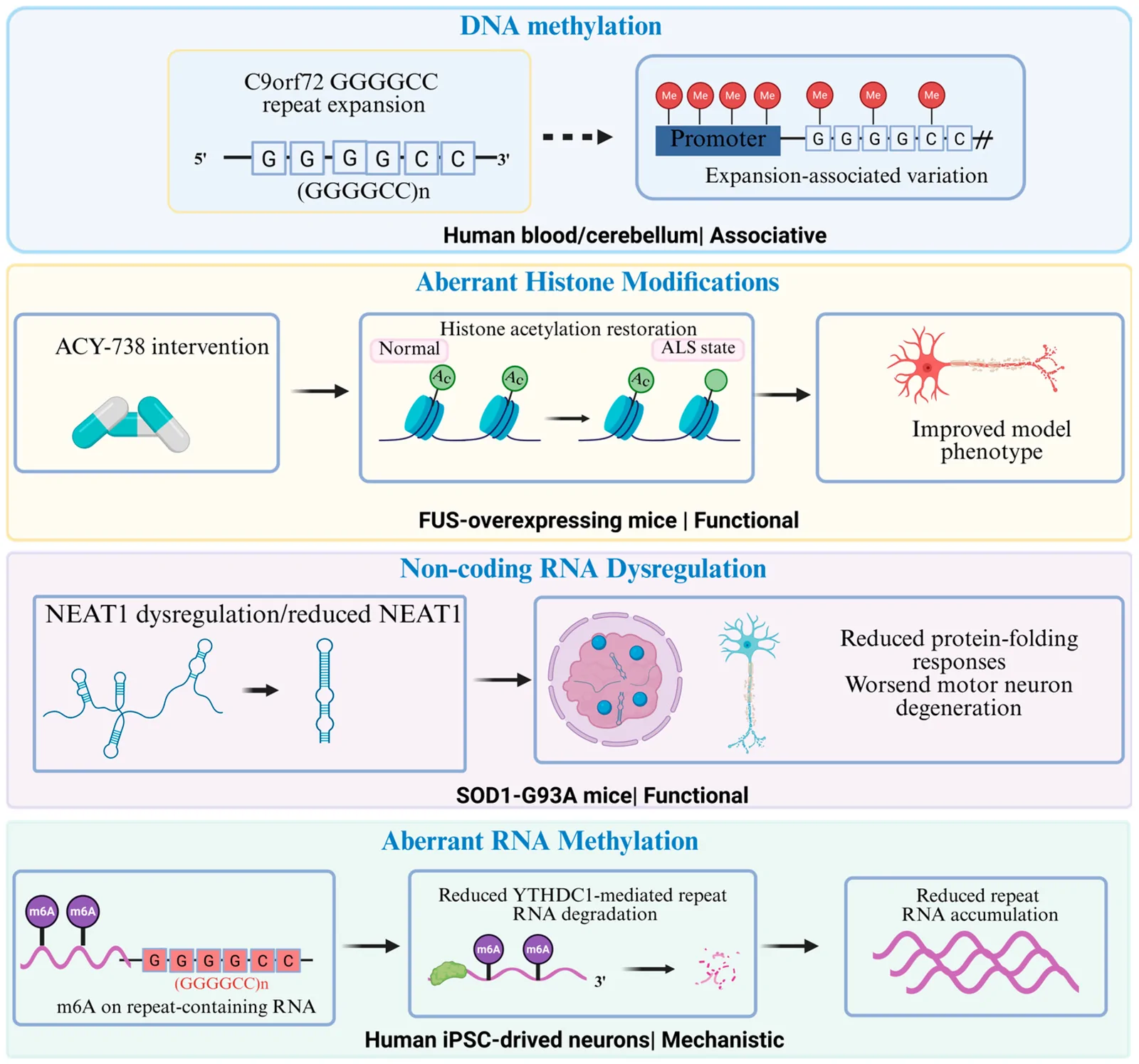
Selected regulatory findings in ALS. Panels show C9orf72 expansion methylation in human samples, ACY-738 intervention in FUS-overexpressing mice, Neat1 deletion in SOD1-G93A mice, and m6A/YTHDC1-dependent repeat RNA decay in human iPSC-derived neurons. Human NEAT1 observations depend on nuclear TDP-43 status. The mouse deletion phenotype is shown separately. C9orf72 findings concern expansion carriers. FTD, frontotemporal dementia; iPSC, induced pluripotent stem cell. Labels distinguish associative observations, functional perturbation evidence, and mechanistic pathway evidence in the indicated study systems. Dashed connections denote association, and solid arrows denote experimentally supported effects. Model findings do not establish causality or therapeutic efficacy in patients. (Created in BioRender. Xing, Li (2026). https://BioRender.com/2fwkvyx, accessed on 13 October 2026).

Epigenetic regulation has been established as a critical regulatory mechanism in the pathogenesis of amyotrophic lateral sclerosis (ALS) through its modulation of gene expression, RNA metabolism, and intracellular homeostasis. Abnormal alterations in epigenetic modifications—including DNA methylation, histone modifications, non-coding RNAs, and RNA methylation—collectively contribute to the development of C9orf72-associated toxicity, protein aggregation, neuroinflammatory activation, synaptic dysfunction, and motor neuron degeneration. The key epigenetic mechanisms involved in ALS progression are summarized in Figure 3.

### 2.4. Chromatin Accessibility and Cell-Type-Specific Regulation

Chromatin accessibility provides a complementary dimension of epigenetic regulation, reflecting the accessibility of genomic regions to regulatory proteins. The assay for transposase-accessible chromatin using sequencing (ATAC-seq) profiles accessible regions, whereas single-nucleus ATAC-seq (snATAC-seq) resolves these patterns across cell populations. These measurements complement DNA methylation and histone profiling rather than serving as interchangeable indicators of transcriptional activity.

In AD, bulk ATAC-seq combined with computational deconvolution has identified cell-type-associated accessibility signatures related to cognitive resilience despite substantial neuropathology [87]. Single-nucleus analyses of postmortem prefrontal cortex have further mapped cell-type-specific regulatory landscapes and reported epigenomic erosion associated with late-stage AD [88]. In PD, parallel snATAC-seq and snRNA-seq analyses of temporal cortex identified altered regulatory-element accessibility and transcriptional networks in a glutamatergic neuronal subtype [89]. As discussed in Section 2.3.2, studies of C9orf72-associated ALS/FTD have also identified context-dependent accessibility and transcriptional changes in motor and frontal cortices [70]. These findings emphasize the importance of cell type, brain region, and genetic background when interpreting disease-associated regulatory changes.

However, increased accessibility does not necessarily demonstrate enhancer activation, increased target-gene expression, or a pathogenic effect. Bulk measurements may reflect differences in cellular composition, while single-nucleus analyses remain sensitive to sparse coverage, tissue quality, and analytical choices. Computational deconvolution, inferred transcription factor activity, and peak-to-gene links require independent validation. Most disease-associated findings from postmortem tissue also lack temporal resolution. Integrated accessibility and transcriptomic profiling, followed by targeted perturbation and rescue experiments, is therefore needed to distinguish pathogenic regulation from compensatory responses or secondary consequences of neuronal injury.

### 2.5. Convergent Pathways and Disease-Associated Mechanisms

Across AD, PD, and ALS, epigenetic alterations may converge on neuroinflammation, mitochondrial and metabolic dysfunction, synaptic abnormalities, and impaired protein homeostasis. However, convergence at the level of downstream pathology does not imply identical upstream mechanisms or uniform directions of epigenetic change. The biological effects depend on the affected cell type, molecular target, disease stage, and experimental system.

Neuroinflammation provides one point of convergence. In AD and PD, the studies discussed above implicate histone modifications and non-coding RNAs in regulating microglial metabolic and inflammatory programs, including H4K12 lactylation in AD and H3K9 lactylation and miR-218-5p-related signaling in PD [20,46,48]. In ALS, blood methylation studies identify associations with immune and inflammatory pathways [61,67], although these peripheral observations do not establish the corresponding mechanisms within the central nervous system. Thus, the three diseases share an inflammatory context, but the cellular sources and strength of mechanistic evidence are different.

Mitochondrial and metabolic disturbances also intersect with epigenetic regulation. PD studies connect mitochondrial stress with H3K27ac dysregulation and altered enhancer activity [42], whereas restoration of histone acetylation in a FUS-overexpression mouse model ameliorates metabolic abnormalities and motor deficits [68]. Synaptic vulnerability provides a further common endpoint. Neuronal METTL3 depletion produces postsynaptic injury in experimental AD-related studies [29], while muscle-derived miR-126 regulates local TDP-43 synthesis and neuromuscular junction integrity in ALS models [75]. These findings indicate convergence on neuronal connectivity and highlight differences between central synaptic dysfunction and neuromuscular junction degeneration.

Impaired protein homeostasis connects these shared pathways to characteristic disease-associated substrates. In AD, the mechanisms reviewed predominantly involve Aβ metabolism, tau regulation, and synaptic protein turnover. In PD, several regulatory pathways converge on SNCA/α-synuclein expression and clearance, including the circ-Pank1–miR-7a-5p pathway and METTL14-dependent m6A regulation [52,55]. In ALS, prominent examples involve TDP-43/FUS-associated RNA regulation and, within C9orf72 expansion-associated ALS/FTD, repeat RNA processing and DPR production [75,77,79,81]. These mechanisms are preferentially linked to the characteristic pathology of each disease rather than being exclusive to it.

This comparison also argues against uniform therapeutic manipulation of an epigenetic mark across diseases. For example, increased H3K27ac is reported in PD brain tissue, whereas reduced global histone acetylation occurs in the FUS-overexpression mouse model [41,68]. These observations concern different marks, tissues, and experimental contexts and therefore cannot be treated as a direct contradiction. Similarly, the effects of m6A regulation vary across neuronal and immune-cell populations [29,31]. Translational strategies should consequently prioritize defined molecular targets and cell populations rather than assume that globally increasing or decreasing an epigenetic modification will produce consistent benefits.

## 3. Candidate Epigenetic Biomarkers: Evidence, Validation, and Potential Applications

Epigenetic alterations in neurodegenerative diseases show disease-specific patterns, but they also involve several shared layers of molecular regulation. To give a clearer overview of these features, this section focuses on AD, PD, and ALS. It mainly reviews four major regulatory levels: DNA methylation, histone modifications, non-coding RNAs, and RNA methylation, with particular attention to m6A modification.

The biomarkers discussed below should be regarded as candidates at different stages of validation rather than established clinical tests. Their evaluation requires discrimination not only between patients and healthy controls but also from clinically relevant disease mimics and other neurodegenerative disorders. Where reported, diagnostic performance should include sensitivity, specificity, predictive values, and reproducibility across independent cohorts and laboratories. AUC values alone do not establish clinical utility, and predictive values depend on disease prevalence in the intended population. Incremental value should also be assessed against established clinical, imaging, and molecular approaches. Age, sex, medication exposure, disease stage, comorbidities, and sample-processing procedures may influence biomarker measurements, which should be considered in study design and interpretation.

Disease-associated molecular alterations are not automatically biomarker candidates with demonstrated diagnostic or prognostic performance. Where the cited studies do not evaluate discrimination, independent validation, or prospective prediction, the findings are described as disease-associated regulatory features whose biomarker relevance remains exploratory.

### 3.1. Epigenetic Markers in AD

#### 3.1.1. DNA Methylation Markers

DNA methylation abnormalities in AD mainly affect genes involved in Aβ metabolism, Tau pathology, and neuroinflammation. For example, altered promoter methylation of genes such as sortilin-related receptor 1 (SORL1) and sirtuin 1 (SIRT1) may influence their expression and thereby participate in Aβ production and clearance [10]. In addition, epigenetic clock studies based on multiple CpG sites have shown that acceleration of DNA methylation-based PhenoAge (DNAmPhenoAge) and DNA methylation-based GrimAge (DNAmGrimAge) is associated with cognitive decline, cortical atrophy, and white matter lesion burden. These findings suggest that DNA methylation is not only involved in disease development but may also reflect biological aging and disease progression in AD [17].

#### 3.1.2. Histone Modification Markers

Several histone modifications are altered in AD, among which active marks such as H3K4me3, H3K9ac, and H3K27ac are closely related to neuronal gene regulation. Changes in H3K4me3 at the ankyrin 1 (ANK1) locus have been reported to correlate with AD pathology and cognitive decline [90]. These abnormal histone modifications may contribute to neuronal dysfunction and disease-related transcriptional network remodeling by altering chromatin accessibility and transcriptional activity.

#### 3.1.3. Non-Coding RNA Markers

Altered miR-129-5p expression in postmortem brain tissue is associated with amyloid and tau pathology and cognitive decline. These findings support disease relevance but do not establish its performance as an accessible diagnostic or prognostic biomarker [91]. In addition, some lncRNAs and circRNAs are involved in neuroinflammation, synaptic plasticity, and neuronal survival, indicating that non-coding RNAs may reflect AD-related pathological changes at the post-transcriptional level.

#### 3.1.4. RNA Methylation (m6A) Markers

m6A modification is one of the most widely studied epitranscriptomic markers in AD. Reduced METTL3 expression and disrupted m6A modification may influence neuronal homeostasis, aberrant cell-cycle re-entry, synaptic function, and cognitive behavior [29]. Meanwhile, abnormal m6A regulation is also associated with neuroinflammation and impaired proteostasis, suggesting that RNA methylation may participate in several key pathological processes in AD.

### 3.2. Epigenetic Markers in PD

#### 3.2.1. DNA Methylation Markers

SNCA intron 1 hypomethylation has been observed in postmortem frontal cortex from individuals with GBA1-associated PD. This represents a subgroup-associated epigenetic feature, and its diagnostic sensitivity, specificity, and applicability to broader PD populations remain to be established [35]. In addition, genome-wide methylation studies have identified widespread differentially methylated sites in the peripheral blood of patients with PD. These sites are mainly related to immune and inflammatory responses, mitochondrial function, and environmental exposure. This suggests that DNA methylation may partly reflect PD-related disease risk and pathological heterogeneity.

#### 3.2.2. Histone Modification Markers

Histone modification abnormalities in PD are mainly reflected in increased active acetylation marks, especially H3K27ac. Studies have shown abnormal enhancer and promoter activation at PD-related gene loci, including SNCA, PARK7, PRKN, and MAPT [41]. These changes may disturb disease-related gene expression and further contribute to dopaminergic neurodegeneration and neuroinflammatory responses.

#### 3.2.3. Non-Coding RNA Markers

miRNAs, lncRNAs, and circRNAs are all dysregulated to varying degrees in PD. miR-7 can regulate α-synuclein expression and neuroinflammatory responses, and its downregulation may promote the pathological spread of α-synuclein [47]. lncRNA HOTAIRM1 can regulate α-synuclein expression through the HOTAIRM1/miR-223-3p/α-syn axis [50], whereas circ-Pank1 aggravates dopaminergic neuronal injury through the miR-7a-5p/α-syn pathway [52]. These molecules are candidate indicators, but their detectability, diagnostic specificity, and stratification value in liquid-biopsy samples require independent validation.

#### 3.2.4. RNA Methylation (m6A) Markers

m6A dysregulation in PD mainly involves the METTL14–m6A–YTHDF2 regulatory axis. Reduced METTL14 expression can alter m6A modification of α-syn mRNA and decrease its degradation efficiency, thereby promoting α-synuclein accumulation [55]. In addition, m6A abnormalities may affect mitochondrial function, oxidative stress, and neuroinflammation, suggesting that RNA methylation participates in multilayered pathological regulation in PD.

### 3.3. Epigenetic Markers in ALS

#### 3.3.1. DNA Methylation Markers

DNA methylation abnormalities in ALS are mainly characterized by widespread differentially methylated sites across the genome. These sites are enriched in pathways related to metabolism, cholesterol biosynthesis, and immune and inflammatory responses, and are associated with disease progression rate and survival [61]. In addition, GrimAge-based epigenetic age acceleration is associated with occupational exposure, sex, and survival, suggesting that DNA methylation may integrate the effects of aging and environmental factors on ALS risk [67].

#### 3.3.2. Histone Modification Markers

In C9orf72-associated ALS/FTD, repressive histone methylation marks, including H3K9me3, H3K27me3, H3K79me3, and H4K20me3, are markedly enriched [71]. These modifications can lead to transcriptional silencing and haploinsufficiency of C9orf72, further affecting RNA metabolism and neuronal survival. This suggests that abnormal histone modifications may represent an important pathogenic mechanism in ALS.

#### 3.3.3. Non-Coding RNA Markers

Non-coding RNAs in ALS are closely linked to TDP-43 pathology and neuromuscular junction damage. Reduced levels of muscle-derived extracellular vesicle miR-126a-5p may promote aberrant local synthesis and accumulation of TDP-43 in axons and neuromuscular junctions [75]. Dysregulated expression of lncRNA NEAT1 may influence paraspeckle homeostasis and cellular stress responses [77], while C9orf72 repeat expansion-associated circular RNA can serve as an abnormal translation template to produce dipeptide repeat proteins, thereby contributing to both RNA toxicity and protein toxicity [79].

#### 3.3.4. RNA Methylation (m6A) Markers

These findings support a role for m6A-dependent RNA regulation in the studied C9orf72-associated ALS/FTD models. Their relevance to clinically measurable biomarkers, including diagnostic discrimination and prospective prognostic performance, remains to be established [81]. Overall, RNA methylation abnormalities may reflect RNA metabolic imbalance and neuronal degeneration at the epitranscriptomic level in ALS, making them an emerging class of epigenetic markers that have received increasing attention in recent years.

Peripheral blood and other liquid-biopsy samples offer practical access to candidate biomarkers, but their ability to represent CNS epigenetic alterations requires independent validation. Blood methylation profiles may reflect leukocyte composition and systemic inflammatory or metabolic states rather than changes within vulnerable neurons. Circulating RNAs and extracellular vesicles may originate from multiple tissues, while CSF measurements do not necessarily identify the contributing cell population or brain region. Collection, processing, storage, RNA extraction, assay platform, and normalization procedures may further affect the results. Consequently, exploratory associations with disease status should not be interpreted as established diagnostic or prognostic utility. Paired peripheral–CNS studies, standardized procedures, independent replication, and prospective evaluation in clinically representative populations are needed.

In addition, we provide a brief summary of the four major categories of epigenetic biomarkers identified in AD, PD, and ALS, as shown in Table 1.

## 4. Epigenetic and Complementary RNA-Targeted Therapeutic Strategies

The therapeutic approaches discussed in this section comprise two related but conceptually distinct categories. Conventional epigenetic therapies directly modulate chromatin- or RNA-modifying systems, including DNA methylation, histone modifications, and RNA methylation [11,92]. By contrast, sequence-specific RNA-targeted therapies, such as antisense oligonucleotides (ASOs) and small interfering RNAs (siRNAs), directed against MAPT, SNCA, or SOD1, primarily alter the abundance or translation of selected transcripts and are not necessarily epigenetic therapies in the conventional sense. They are considered as complementary gene-regulatory strategies because they act downstream of, or in parallel with, the regulatory networks reviewed above and provide an important benchmark for the clinical translation of mechanism-based molecular therapies. Most direct epigenetic interventions remain at the preclinical or early translational stage, whereas selected RNA-targeted therapies have progressed further clinically [11,93].

Throughout this section, findings from cellular and animal models are distinguished from evidence obtained in human studies. Improvements in experimental pathology, behavior, or survival provide preclinical support but do not establish therapeutic efficacy in patients. Likewise, target-related biomarker changes in a clinical study should be distinguished from demonstrated benefits in symptoms, function, or survival.

### 4.1. DNA Methyltransferase Inhibitors

Aberrant DNA methylation plays a widespread role in regulating the expression of disease-associated genes in Alzheimer’s disease (AD), Parkinson’s disease (PD), and amyotrophic lateral sclerosis (ALS). Consequently, therapeutic strategies targeting DNA methyltransferases (DNMTs) have emerged as one of the earliest explored avenues for epigenetic intervention. Classic DNMT inhibitors—including 5-azacytidine, decitabine (5-aza-2′-deoxycytidine), and RG108—can reduce levels of aberrant DNA methylation by inhibiting DNMT activity, thereby restoring the expression of genes that have been epigenetically silenced. Currently, these drugs are primarily employed in mechanistic studies to investigate whether abnormal DNA methylation states are reversible [92,94].

In AD, DNMT inhibitors are currently predominantly utilized in fundamental experimental investigations. These pharmacological agents modulate Aβ metabolism, neuronal function, and the expression of neuroinflammation-related genes by downregulating aberrant DNA methylation levels. Nevertheless, existing DNMT inhibitors generally lack both site and cell-type specificity. This deficiency can induce global transcriptional dysregulation across the entire genome and impair normal neural plasticity processes, which consequently creates substantial barriers to their clinical translation [92,94].

In PD, existing studies on DNA methylation regulation are predominantly concentrated on the pathological pathways mediated by the SNCA gene and its encoded product α-synuclein. Current research evidence indicates that the DNA methyltransferase (DNMT) inhibitor 5-aza-2′-deoxycytidine (decitabine) can reduce the overall level of genomic DNA methylation, which in turn upregulates the transcriptional expression of pathogenic genes associated with PD. Nevertheless, non-specific widespread DNA demethylation will further enhance the expression of PD-related pathogenic genes and aggravate cytotoxic damage to dopaminergic neurons, suggesting that non-selective DNMT inhibition strategies carry non-negligible potential adverse effects. Therefore, in contrast to genome-wide non-specific methylation intervention, targeted and precise regulation of disease-specific differentially methylated sites has emerged as a critical research direction for future investigation in this field [95].

In ALS, the efficacy of DNA methylation-targeted therapy has been partially supported by experimental evidence from animal studies. Research by Martin et al. demonstrated that following 5-azacytidine intervention, abnormally elevated DNA methylation levels in the spinal cord and skeletal muscle tissues of hSOD1-G93A ALS model mice were significantly reduced, with improved motor function and markedly prolonged survival, suggesting that correcting DNA methylation abnormalities may represent a potential therapeutic strategy for ALS [63]. However, to date, this approach lacks robust clinical trial evidence in humans, and its safety and efficacy require further systematic evaluation.

### 4.2. Histone Deacetylase Inhibitors

Histone modifications constitute another critical category of epigenetic therapeutic targets. Among these modifications, histone acetylation status is dynamically modulated by two classes of enzymes: histone acetyltransferases (HATs) and histone deacetylases (HDACs). HDAC inhibitors are capable of upregulating histone acetylation levels, facilitating the conformational transition of chromatin to an open state, and consequently upregulating the expression of neuroprotection-associated genes [96,97].

In AD, various histone deacetylase inhibitors—including sodium butyrate, suberoylanilide hydroxamic acid (SAHA), and valproic acid (VPA)—have demonstrated potential neuroprotective effects in experimental disease models. These agents can modulate synaptic plasticity, improve learning and memory functions in models, and mitigate pathology mediated by Aβ and Tau proteins. However, given the functional heterogeneity among different HDAC family members, broad-spectrum HDAC inhibition may be linked to nonspecific effects, and clinical translation of such drugs remains limited by issues such as poor selectivity and long-term safety concerns [96,97].

In PD, current research on histone deacetylase inhibition strategies primarily focuses on four core directions: regulating α-synuclein toxicity, modulating autophagic activity, maintaining mitochondrial physiological function, and preserving the survival of dopaminergic neurons. Among these directions, the selective HDAC6 inhibitor Tubastatin A has exhibited significant neuroprotective efficacy in both cellular and animal models of PD. Mechanistically, HDAC6 inhibition reduces α-synuclein-mediated neurotoxicity, improves intracellular protein homeostasis, and exerts a protective effect on dopaminergic neurons [98]. In addition, sodium butyrate and valproic acid can potentially exert neuroprotective effects by restoring histone acetylation levels and regulating inflammation-related signaling pathways, but their therapeutic value for PD treatment still requires further rigorous verification [96,97].

In ALS, restoring histone acetylation levels also holds potential therapeutic value. In mouse models of ALS associated with FUS mutations, the HDAC inhibitor ACY-738, which can cross the blood–brain barrier, restored whole-genome histone acetylation levels, improved metabolic abnormalities and motor phenotypes in model animals, and prolonged their survival [68]. However, existing clinical studies have yielded inconsistent results: randomized trials in ALS patients demonstrated that valproic acid did not significantly prolong survival or delay disease progression, suggesting the need for developing targeted drugs with higher selectivity and better safety profiles against specific HDAC subtypes in this field [99].

The reported benefits of HDAC inhibitors in AD and PD are primarily derived from preclinical models and should not be interpreted as established efficacy in patients [96,97,98]. In ALS, ACY-738 improved outcomes in a FUS-overexpression mouse model [68], whereas the randomized valproic acid trial did not demonstrate improved survival or disease progression [99]. These findings highlight the distinction between preclinical and clinical benefit. They also indicate that results obtained with one compound or model cannot be generalized to the entire HDAC inhibitor class.

### 4.3. RNA Expression-Targeted Therapies and Non-Coding RNA Modulation

The approaches discussed in this subsection comprise direct transcript-targeting therapies and non-coding RNA modulation. ASOs targeting MAPT, APP, SNCA, or SOD1 primarily lower specific transcripts, and C9orf72-directed ASOs may target repeat-containing RNAs. These interventions are included as complementary RNA-targeted strategies, and their clinical results do not constitute evidence for epigenetic therapeutic efficacy. By contrast, miRNA mimics, antagomirs, and lncRNA-directed interventions modulate non-coding RNA pathways, although their classification as epigenetic interventions depends on the demonstrated mechanism. The ncRNA-directed approaches discussed here remain preclinical in AD, PD, and ALS [93,100].

In AD, RNA-targeted therapeutic strategies predominantly focus on MAPT and APP. Among these approaches, MAPTRx/BIIB080 is an antisense oligonucleotide designed to reduce MAPT RNA and tau production. In a randomized, placebo-controlled phase 1b multiple-ascending-dose study, MAPTRx/BIIB080 was evaluated primarily for safety, with pharmacokinetics assessed as a secondary endpoint and cerebrospinal-fluid tau concentrations assessed as exploratory target-engagement measures. Treatment produced dose-dependent reductions in cerebrospinal-fluid total tau and phosphorylated tau and was generally tolerated during the study. However, the trial was not designed or powered to establish clinical efficacy, and the observed biomarker reductions should not be interpreted as evidence of clinical benefit [101]. APP-targeting ASOs and miRNA-based approaches remain preclinical and are being investigated for effects on amyloid-beta metabolism, tau phosphorylation, and neuroinflammatory pathways [93,100].

In PD, RNA-targeted therapeutic research primarily focuses on SNCA and alpha-synuclein. SNCA-targeting ASOs are sequence-specific transcript-lowering therapies. They bind to SNCA mRNA to promote its degradation, thereby reducing alpha-synuclein production rather than directly modifying an epigenetic state. In cellular and animal models, these ASOs reduce alpha-synuclein aggregation and fibril-induced pathological propagation, protecting dopaminergic neurons [102,103]. SNCA-targeting siRNAs and miRNA-mediated strategies remain exploratory, with delivery efficiency and long-term safety representing major translational barriers [93,100].

In ALS, RNA-targeted therapy has reached clinical application for a genetically defined subgroup. Tofersen is a sequence-specific RNA-targeted precision therapy, rather than a conventional epigenetic drug, and is included here as a clinically advanced comparator for gene-regulatory intervention. It binds to SOD1 mRNA to reduce SOD1 protein as well as plasma neurofilament light chain. In the 28-week randomized trial, however, tofersen did not significantly improve the prespecified primary clinical endpoint, although exploratory extension analyses favored earlier treatment [104]. It received accelerated approval for SOD1-associated ALS on the basis of neurofilament reduction, a surrogate endpoint considered reasonably likely to predict clinical benefit, while confirmatory evidence of clinical benefit remains required [105]. Other RNA-targeted strategies for C9orf72, TDP-43/FUS-related pathways, and miRNA networks remain preclinical [93,100].

### 4.4. Direct Epitranscriptomic Therapy Targeting RNA Methylation (m6A)

Compared with DNA methylation and histone-modifying therapies, direct epitranscriptomic drug development targeting N6-methyladenosine (m6A) remains at an early stage. Unlike ASOs or siRNAs, which lower selected transcripts through sequence recognition, these agents directly modulate RNA-modification machinery. The m6A system includes methyltransferase writers such as METTL3/METTL14, demethylase erasers such as FTO and ALKBH5, and reader proteins that regulate RNA stability, translation, and degradation [106,107].

Small-molecule modulation of m6A machinery has been investigated primarily in oncology. STM2457 showed anti-leukaemic activity in preclinical models [108], whereas STC-15 has undergone phase I evaluation in patients with advanced malignancies. The cited STC-15 report is a conference abstract describing early safety, pharmacokinetic, pharmacodynamic, and exploratory clinical findings [109]. It does not establish blood–brain barrier penetration, target engagement in disease-relevant CNS cells, efficacy in AD, PD, or ALS, or long-term neurological safety. These findings should therefore be presented as oncology evidence rather than validation of m6A-directed treatment of neurodegenerative disease. FTO inhibitors such as FB23 and FB23-2 have also been investigated in tumour and inflammatory models, which similarly require further evaluation in neurodegenerative settings [106,110,111].

METTL3 and METTL14 also support physiological RNA regulation and neuronal homeostasis. Cholinergic neuron-specific Mettl3 depletion caused motor neuron loss and neuromuscular degeneration in mice [84], emphasizing that broad suppression of m6A writers may carry neurological risks. Genetic depletion does not directly predict the effects of partial pharmacological inhibition, but it makes cell specificity, CNS exposure, dose–response relationships, and a tolerable treatment window essential considerations [84,85].

Changes in METTL3, METTL14, or FTO expression do not alone establish corresponding changes in catalytic activity, global m6A abundance, or modification of a particular transcript. Global measurements may also obscure opposite changes among individual RNAs or cell populations. The functional consequences depend on the modified transcript and site, reader proteins, and downstream effects on RNA stability, translation, or processing. Accordingly, neither increased nor decreased m6A should be interpreted as uniformly pathogenic or protective, and therapeutic intervention requires target-specific functional validation.

At present, m6A-targeted therapeutic agents have not yet been applied in clinical intervention for AD, PD and ALS. A growing body of research indicates that aberrant m6A modification is implicated in multiple pathological processes in neurodegenerative diseases, including neuronal dysfunction, abnormal aggregation of pathological proteins, neuroinflammation and RNA metabolic dyshomeostasis. Accordingly, regulating the catalytic activity of core m6A modification enzymes such as METTL3, FTO and ALKBH5 is considered a promising intervention strategy for restoring RNA homeostasis in the nervous system. Before such strategies can be translated into clinical applications, the disease-specific functional mechanisms of these targets, their delivery efficiency across the blood–brain barrier, and the safety of long-term administration need to be further elucidated [106,107].

These context-dependent findings argue against uniformly increasing or decreasing m6A across neurodegenerative diseases. Therapeutic modulation of METTL3/METTL14 or FTO should therefore be guided by the affected cell population, target RNAs, and disease subtype.

### 4.5. Challenges in Clinical Translation

Although epigenetic therapeutic strategies provide new therapeutic opportunities for AD, PD, and ALS, their clinical translation still faces several important challenges. The blood–brain barrier remains one of the major obstacles, as it limits the entry of many DNMT inhibitors, HDAC inhibitors, and RNA-targeted agents into the central nervous system. Accordingly, future studies should focus on improving delivery efficiency through approaches such as intrathecal administration, viral vectors, lipid nanoparticles, exosomes, or receptor-mediated transport [112,113].

Broad or nonspecific epigenetic manipulation may alter transcription beyond the intended disease-related targets, disrupt physiological neuronal functions such as synaptic plasticity and stress responses, and affect peripheral tissues, all of which are potential risks requiring evaluation. Therapeutic development should therefore establish the relevant target, cell population, and tissue distribution, together with dose–response relationships and a tolerable treatment window.

CNS delivery should be evaluated separately from target engagement. Detectable drug exposure in the brain or CSF does not, by itself, demonstrate activity in the intended cells, and a change in global methylation or acetylation may not confirm modulation of the disease-relevant target. Studies should connect drug exposure with target-related pharmacodynamic effects and functional outcomes. Repeated-dose investigations should additionally assess systemic adverse effects, persistence and reversibility of epigenetic changes, and long-term effects on neuronal and immune homeostasis.

Overall, direct epigenetic therapies, including DNMT inhibitors, HDAC inhibitors, and RNA-methylation modulators, should be distinguished from complementary RNA-targeted therapies such as ASOs, siRNAs, and miRNA-based approaches. These categories act at different levels of gene regulation and differ in target specificity, delivery requirements, translational maturity, and safety considerations. Although both may modify disease-relevant gene-expression states, direct transcript regulation should not be equated with modification of an epigenetic state.

In addition, we provide a brief summary of epigenetic therapeutic strategies for AD, PD and ALS, including representative agents and their current research stages, as shown in Table 2.

## 5. Conclusions, Limitations, and Future Perspectives

### 5.1. Conclusions

In summary, the onset and progression of neurodegenerative diseases cannot be fully explained by genetic factors alone. Genetic studies have identified many pathogenic or risk genes associated with AD, PD, and ALS, including APP, PSEN1/2, SNCA, LRRK2, C9orf72, and SOD1. However, most patients still develop sporadic or late-onset forms of disease. There are also clear differences among patients in age at onset, clinical manifestations, disease progression, and treatment responses. These features indicate that genetic background represents only one part of disease susceptibility. Aging, environmental exposure, metabolic disturbance, inflammatory responses, and cellular stress may also shape disease trajectories through complex molecular regulatory networks [11,114]. In this context, epigenetics provides an important complement to traditional genetic explanations. DNA methylation, histone modifications, non-coding RNA regulation, and RNA methylation can respond to changes in environmental conditions and cellular states, while also producing relatively lasting effects on gene expression without altering the DNA sequence itself [9,114].

This review integrates evidence showing that AD, PD, and ALS have distinct pathological features but share alterations across multiple epigenetic regulatory layers. In AD, these alterations are associated with amyloid-beta/tau pathology, synaptic injury, and neuroinflammation; in PD, they are associated with alpha-synuclein pathology, mitochondrial dysfunction, impaired autophagy, and dopaminergic neuronal degeneration; and in ALS, they are associated with C9orf72 repeat expansion, TDP-43/FUS pathology, RNA dyshomeostasis, neuromuscular-junction injury, and motor-neuron vulnerability [9,95]. Functional interventions in selected cellular and animal models indicate that some epigenetic alterations may contribute to these processes. However, many reported changes remain associative, and their causal, compensatory, or secondary nature has not yet been resolved.

Epigenetic biomarkers and direct epigenetic interventions provide opportunities for clinical translation. DNA methylation signatures, non-coding RNAs, and RNA-modification-related molecules may support risk assessment, diagnosis, monitoring, or patient stratification, although most remain candidate markers requiring further validation [11,92]. DNMT inhibitors, HDAC inhibitors, and RNA-methylation modulators directly target epigenetic or epitranscriptomic regulation. By contrast, ASOs, siRNAs, and related transcript-lowering approaches are complementary RNA-targeted therapies rather than conventional epigenetic treatments. Their relatively rapid progress in ALS and AD nevertheless provides an important translational benchmark for mechanism-based precision therapy [92].

### 5.2. Current Limitations

Despite progress in epigenetic research on AD, PD, and ALS, important limitations remain. Much of the available evidence is observational, making it difficult to determine whether an epigenetic alteration contributes to disease progression, represents a compensatory response, or arises after neuronal injury. This distinction is particularly important for DNA methylation, histone modifications, and RNA methylation, because changes in modification abundance alone do not establish their functional consequences.

Global changes in DNA methylation or histone modifications do not necessarily indicate equivalent changes at individual promoters or enhancers. For example, increased H3K27ac in PD brain tissue and reduced global histone acetylation in a FUS-overexpression mouse model concern different measurements and biological contexts [41,68]. Likewise, altered expression of METTL3, METTL14, FTO, DNMTs, or histone-modifying enzymes does not by itself establish their catalytic activity or target-specific effects. Differences in cellular composition, disease stage, and analytical methods may further contribute to divergent findings.

Another limitation is related to the sample source and the experimental model. Human brain tissue is usually collected after death, which makes it difficult to capture early disease events or dynamic changes during disease progression. Peripheral blood, cerebrospinal fluid and extracellular vesicles are easier to obtain, which may be more practical for clinical tests. However, whether they can accurately reflect the epigenetic changes in the central nervous system still needs to be carefully verified. Epigenetic characteristics may also vary depending on the brain region, cell type and stage of disease. For this reason, bulk tissue analysis may mask cell-type-specific changes in neurons, microglial cells, astrocytes and oligodendrocytes.

The clinical value of many candidate epigenetic biomarkers is still uncertain. AD, PD and ALS all show obvious clinical heterogeneity, and many reported methylation sites, miRNAs, circRNAs or m6A regulators have not been confirmed in large-scale, multi-center, longitudinal cohorts. Differences in sample size, testing platform, statistical threshold and population background may further reduce reproducibility and limit clinical application. Without a standardized workflow and long-term follow-up data, it is still difficult to determine whether these markers have stable diagnostic or prognostic value.

Therapeutic translation is another major challenge. DNMT inhibitors and HDAC inhibitors generally show a wide range of regulatory effects, so they may affect multiple genes, brain regions and cell types at the same time, leading to potential off-target consequences. RNA-targeted therapy usually has higher sequence specificity, but delivery efficiency, immune response, long-term safety and limited applicability to specific patient subgroups are still important issues. RNA methylation regulatory therapy is still mainly limited to mechanistic studies or oncology-related drug development, and the clinical evidence for neurodegenerative diseases is still insufficient. In addition, the blood–brain barrier is still a key obstacle, because many therapeutic drugs need to enter the central nervous system to play their role.

### 5.3. Future Perspectives

Future research should address the specific limitations of current evidence. Because many findings derive from postmortem tissue or cross-sectional studies, longitudinal investigations with repeated sampling are needed to determine whether epigenetic changes precede, accompany, or follow disease progression. Temporal ordering should then be combined with functional experiments rather than treated as sufficient evidence of causality.

The limited independent validation of candidate biomarkers calls for multicenter studies using standardized collection, processing, and analytical procedures. Validation should include clinically relevant disease mimics and assess the reproducibility and incremental value beyond established diagnostic approaches. Because peripheral measurements may not represent CNS alterations, paired-sample investigations should evaluate their biological correspondence where feasible.

Bulk-tissue findings may be confounded by cellular composition. Single-cell and cell-type-specific approaches should therefore distinguish regulatory changes within vulnerable neurons from glial responses or shifts in cell abundance. Multi-omics integration may further help prioritize candidate pathways [115,116]. These candidates require locus- or transcript-specific perturbation, pathway-dependence testing, and rescue experiments to separate pathogenic effects from protective or secondary changes. Epigenetic editing offers an approach for testing selected locus-specific regulatory mechanisms [117]. Joint snATAC-seq and snRNA-seq profiling should link cell-type-specific accessibility changes to transcriptional alterations, followed by targeted perturbation to test inferred regulatory relationships.

Finally, the broad effects and delivery limitations of existing interventions justify prioritizing selective targets and improved CNS delivery. Delivery strategies discussed in the reviewed literature provide a basis for further optimization [112]. Drug exposure should be linked to target engagement in the intended cell population, dose–response relationships, and long-term safety. Clinical studies should evaluate molecular responses alongside clinically meaningful cognitive, functional, or survival outcomes, while specifying the patient populations to which their findings can be applied.

## Figures and Tables

**Table 1:** Summary of selected epigenetic alterations and candidate biomarkers in AD, PD, and ALS, specifying the reported associations or functional effects, supporting evidence levels, and study contexts.

Disease	Biomarker Validation Status and Major Limitations	Potential Translational Implications	Epigenetic Biomarker Categories	Reported Alteration or Candidate Marker	Role in Disease	Evidence Level and Study Context	Ref.
AD	Observational design; clock-dependent results; peripheral origin	Candidate prognostic stratification; requires external validation	DNA methylation	SORL1; SIRT1	Reflects dysregulated expression of genes related to Aβ production, clearance, and cellular stress responses and may help identify early AD-related molecular changes	Primary studies require separate assessment	[10]
				DNAmPhenoAge; DNAmGrimAge	Accelerated DNAmPhenoAge and DNAmGrimAge are associated with cognitive decline, cortical atrophy, white matter disease burden, and an increased risk of progression from normal cognition to MCI or AD	Associative; peripheral blood DNA methylation with longitudinal clinical and MRI assessments in the ADNI cohort	[17]
	Cross-sectional tissue analysis; no locus-specific intervention	Candidate regulatory locus for functional investigation	Histone modification	H3K4me3(ANK1)	Reduced H3K4me3 at the ANK1 locus has been observed in the entorhinal cortex of individuals with a high AD neuropathological burden and is negatively associated with local DNA methylation, suggesting altered transcriptional regulation at this locus	Associative; ChIP-based analysis of human postmortem brain tissue	[90]
	Predicted pathways are not equivalent to validated targets; brain measurements do not establish liquid-biopsy utility	Candidate biomarker requiring accessible-sample validation	Non-coding RNA	miR-129-5p	Reduced miR-129-5p is associated with greater amyloid and Tau pathology and faster cognitive decline; its predicted target genes are enriched in glucose metabolism, ErbB, AMPK, MAPK, mTOR, autophagy, and synaptic signaling pathways	Associative; human postmortem brain miRNA profiling integrated with neuropathology and longitudinal cognitive data	[91]
	Effects depend on cell type; findings do not justify global METTL3 inhibition or activation	Rationale for cell-selective m6A modulation	RNA methylation	METTL3; m6A	Reduced neuronal METTL3 and m6A levels promote aberrant cell-cycle re-entry, oxidative stress, synaptic loss, and neuronal apoptosis, thereby contributing to AD-related neurodegeneration	Associative in human postmortem brain; functional and mechanistic support from neuronal cultures and mouse experiments, including rescue studies	[29]
PD	Observational design; clock-dependent results; peripheral origin	Candidate subgroup-associated epigenetic feature	DNA methylation	SNCA intron 1 hypomethylation; SNCA promoter CpG methylation	Associated with a distinct epigenetic profile in GBA1-associated PD and may alter transcriptional regulation of SNCA, although the study did not directly demonstrate increased SNCA expression or a causal effect on α-synuclein accumulation	Associative; targeted DNA methylation analysis of human postmortem frontal cortex stratified by GBA1 mutation status	[35]
	Bulk-tissue analysis; limited evidence that the observed changes initiate degeneration	Supports investigation of selective chromatin regulation	Histone modification	H3K27ac	H3K27ac hyperacetylation: Genome-wide elevation and abnormal enrichment at active promoters and enhancers, including regions associated with SNCA, PARK7, PRKN, and MAPT, accompanied by disrupted coupling between promoter acetylation and gene expression	Associative for PD-related chromatin changes; human postmortem brain profiling, with complementary sirtuin-inhibition experiments in SH-SY5Y cells	[41]
	Rotenone-model dependence; limited human validation	Preclinical RNA-targeting candidate	Non-coding RNA	miR-7	Suppresses α-synuclein expression, aggregation, and pathological propagation, thereby reducing neuroinflammation and dopaminergic neurodegeneration	Functional; AAV-mediated miR-7 overexpression in an α-synuclein PFF-injected mouse model	[47]
				HOTAIRM1; miR-223-3p	HOTAIRM1 sponges miR-223-3p and relieves its inhibitory effect on α-synuclein, promoting α-synuclein accumulation and neuronal apoptosis	Mechanistic in primary dopaminergic neurons, with mouse-model support; associative findings in human peripheral blood	[50]
				circ-Pank1; miR-7a-5p	circ-Pank1 sequesters miR-7a-5p, increases α-synuclein expression, and may contribute to dopaminergic neuronal degeneration	Mechanistic in rotenone-treated MN9D cells, with mouse-model support; reporter and pathway-reversal experiments	[52]
	Peripheral diagnostic findings and neuronal mechanisms require separate validation	Candidate biomarker and experimental RNA-turnover target	RNA methylation	METTL14; m6A; YTHDF2	Reduced METTL14-mediated m6A modification weakens YTHDF2-dependent degradation of α-synuclein mRNA, thereby increasing α-synuclein stability and accumulation and contributing to PD-related neurodegeneration	Associative in human PBMCs; mechanistic support from in vitro RNA-binding, RNA-stability, and gene-perturbation experiments	[55]
ALS	Tissue specificity; residual confounding and reverse causation	Candidate risk/prognostic signatures; individual sites require functional validation	DNA methylation	Genome-wide differentially methylated sites	ALS-associated blood DMPs, particularly methylation changes at DHCR24, ABCG1, MSMO1, and SLC7A11, which are linked to cholesterol metabolism, immunity, oxidative stress, and patient survival	Associative; large-scale whole-blood EWAS meta-analysis with survival analyses	[61]
				DNAmGrimAge	Reflects accelerated biological ageing and is associated with ALS status, occupational exposures, and shorter survival, particularly in male patients	Associative; blood-based case–control study with survival, occupational-exposure, and immune-cell analyses	[67]
	Overexpression-model generalizability; intervention does not isolate a single histone locus	Preclinical rationale for selective, CNS-accessible HDAC modulation	Histone modification	H3K9me3; H3K27me3; H3K79me3; H4K20me3	Enrichment of H3K9me3, H3K27me3, H3K79me3, and H4K20me3 at the expanded C9orf72 locus may contribute to heterochromatin formation and transcriptional silencing, resulting in reduced C9orf72 expression; H3K9me3 and H3K27me3 associated with the expanded allele are also detectable in peripheral blood	Associative in human brain and blood; functional support from 5-aza-dC treatment of patient-derived fibroblasts	[71]
	Generalizability across ALS subtypes and delivery feasibility require evaluation	Candidate approach to preserve NMJs through muscle–neuron communication	Non-coding RNA	miR-126a-5p	Muscle-derived EV miR-126a-5p suppresses aberrant local translation and accumulation of TDP-43 at the NMJ, thereby preserving axonal protein synthesis and NMJ integrity	Mechanistic; primary and human iPSC-derived neuromuscular co-cultures and SOD1G93A mice, with complementary patient observations	[75]
				NEAT1	Loss of nuclear TDP-43 reduces NEAT1 expression and weakens its protective function, thereby exacerbating motor neuron degeneration	Associative in human postmortem spinal cord; mechanistic support for TDP-43-dependent transcription and functional evidence from Neat1 knockout in hSOD1G93A mice	[77]
				C9orf72 repeat expansion-associated circular RNA	Stable circular repeat RNA is exported to the cytoplasm and translated into DPRs, promoting RNA/protein toxicity and ALS/FTD progression	Mechanistic; cellular reporter and single-molecule imaging experiments, with RNA-export perturbation in patient iPSC-derived neurons	[79]
	Mainly C9orf72-associated ALS/FTD; clinical efficacy and safety remain unestablished	Genotype-informed preclinical RNA-homeostasis strategy	RNA methylation	m6A; YTHDC1; METTL3; METTL14	Global m6A reduction caused by decreased METTL3/METTL14 impairs YTHDC1-mediated degradation of C9ORF72 repeat RNA and disrupts YTHDF2-dependent mRNA turnover, leading to repeat RNA and DPR accumulation, transcriptome-wide RNA dysregulation, and neurodegeneration	Associative in human postmortem brain; mechanistic evidence from patient iPSC-derived neurons and cellular reporters, including m6A-restoration experiments	[81]

**Table 2:** Therapeutic drugs and strategies for AD/PD/ALS.

Disease	Therapeutic Strategy	Drugs	Research Stage	Mechanistic Candidate, Clinical Diagnostic Performance not Established by the Cited Study	Ref.
AD	Inhibition of DNA methyltransferase	5-azacytidine	Mainly in vitro studies and animal models (preclinical studies)	Reduces aberrant DNA methylation and restores the expression of epigenetically silenced genes. May regulate AD-associated pathways involved in amyloid-β metabolism, neuronal function, and neuroinflammation	[92,94]
		Decitabine	Mainly in vitro studies and animal models (preclinical studies)	Relieves DNA methylation-mediated transcriptional repression and restores the expression of disease-associated genes, potentially improving AD-related molecular abnormalities	[92,94]
		RG108	Primarily in vitro cellular studies	Reduces abnormal DNA methylation with relatively lower cytotoxicity compared with nucleoside analog inhibitors. It is mainly used as a research tool to explore whether restoration of DNA methylation homeostasis can improve AD-related molecular abnormalities	[92,94]
	Inhibition of histone deacetylase	Sodium butyrate	Mainly investigated in vitro and in AD animal models (in vivo preclinical studies)	Enhances chromatin accessibility and promotes the expression of neuroprotective genes. Sodium butyrate improves synaptic plasticity, learning and memory performance, and reduces Aβ-associated neurotoxicity in AD models	[96,97]
		Suberoylanilide hydroxamic acid	Mainly investigated in vitro and animal models (in vivo preclinical studies)	Restores abnormal chromatin states, promotes neuronal gene transcription, improves synaptic dysfunction, and shows potential effects in reducing AD-related pathological changes and cognitive impairment	[96,97]
		Valproic acid	Cellular studies and animal models; clinically used for other neurological disorders but not approved for AD treatment	Enhances the expression of neuroprotective genes, improves synaptic function, and reduces Aβ-associated neuronal damage in experimental AD models. However, its clinical translation is limited by insufficient selectivity and potential long-term safety concerns	[96,97]
	Complementary RNA-targeted therapy	MAPTRx/BIIB080 (Tau-targeting ASO)	Phase 1b randomized, placebo-controlled multiple-ascending-dose study	Sequence-specific reduction in MAPT RNA produced dose-dependent decreases in cerebrospinal-fluid total tau and phosphorylated tau, supporting target engagement. The study primarily evaluated safety and was not designed or powered to demonstrate clinical efficacy	[93,101]
		APP-targeting ASOs	Mainly in vitro cellular studies and animal models (preclinical studies)	Reduces APP expression, decreases amyloidogenic processing, and lowers Aβ production, thereby potentially alleviating amyloid-related pathology	[93,100]
		miRNA mimics	Mainly in vitro and animal models (preclinical studies)	Restores the function of downregulated protective miRNAs and regulates post-transcriptional networks involved in Aβ metabolism, Tau phosphorylation, synaptic dysfunction, and neuroinflammation	[100]
PD	DNA methylation modulation	5-Aza-2′-deoxycytidine	In vitro cellular study (dopaminergic neuron model)	Treatment with 5-aza-dC reduces DNA methylation and may be linked to transcriptional activation of PD-associated genes. However, instead of providing neuroprotection, excessive DNA demethylation increases the expression of PD-related genes and exacerbates dopaminergic neuronal toxicity, suggesting that non-selective DNA methylation inhibition may have detrimental effects in PD	[92,95]
		5-Azacytidine	Mainly in vitro and animal preclinical studies in neurological disease models (not PD-specific clinical studies)	Reduces aberrant DNA methylation and restores epigenetically silenced gene expression. In PD, DNMT inhibition is considered a potential strategy to modulate methylation abnormalities affecting genes such as SNCA, although its therapeutic value remains uncertain due to lack of specificity	[92]
		RG108	Mainly in vitro cellular studies	Provides a tool to investigate whether abnormal DNA methylation can be reversed with lower cytotoxicity than nucleoside analog inhibitors. Its potential application in PD remains experimental	[92]
	Inhibition of histone deacetylase	Tubastatin A	In vitro cellular models and in vivo animal models	HDAC inhibitors regulate α-synuclein toxicity, autophagy, mitochondrial function, and dopaminergic neuronal survival. HDAC6 inhibition has shown neuroprotective effects in α-synuclein models, but PD-specific clinical translation remains unavailable	[98]
		Sodium butyrate	Mainly investigated in vitro and animal models (preclinical studies)	Restores abnormal histone acetylation patterns, promotes expression of neuroprotective genes, and may improve oxidative stress, inflammatory responses, and neuronal survival in PD models	[96,97]
		Valproic acid	In vitro and animal studies; clinically used for other neurological disorders but not approved for PD treatment	Modulates chromatin states, regulates neuroprotective and inflammatory pathways, and may improve neuronal function. However, its therapeutic efficacy in PD remains unclear	[96,97]
	Complementary RNA-targeted therapy	SNCA-targeting ASOs (α-synuclein ASOs)	In vitro cellular models and in vivo animal models	ASOs bind to SNCA mRNA and promote its degradation, thereby reducing α-synuclein synthesis. Lower α-synuclein levels decrease α-synuclein aggregation, limit fibril-induced pathological propagation, protect dopaminergic neurons, and improve PD-related pathological changes	[93,100,102,103]
		SNCA-targeting siRNAs	Mainly in vitro and animal studies (preclinical)	Uses RNA interference mechanisms to selectively reduce SNCA expression, decrease α-synuclein production, and potentially alleviate α-synuclein-related toxicity. However, delivery efficiency and long-term safety remain major challenges	[93,100]
		miRNA mimics or miRNA modulators	Mainly in vitro and animal studies	Restores protective miRNA activity or regulates pathogenic RNA networks to reduce α-synuclein expression and improve neuronal homeostasis	[100]
ALS	Inhibition of DNA methyltransferase	5-Azacytidine (5-aza-C)	In vivo ALS mouse model (hSOD1-G93A)	Reduces aberrant DNA methylation in the spinal cord and skeletal muscle, restores epigenetic regulation, improves motor function, and prolongs survival, suggesting DNA methylation as a therapeutic target in ALS	[63]
	Inhibition of histone deacetylase	ACY-738	In vivo FUS-ALS mouse model	Restores global histone acetylation, normalizes metabolic gene expression, improves motor phenotype, and extends survival by correcting epigenetic and metabolic abnormalities	[68]
		Valproic acid	Human clinical trial (ALS patients)	Increases histone acetylation and shows antioxidant and anti-apoptotic properties; however, it fails to improve survival or slow disease progression in ALS patients	[99]
	Complementary RNA-targeted therapy	Tofersen (BIIB067)	Phase 3 randomized trial; open-label extension; accelerated clinical approval based on a surrogate biomarker endpoint	Reduced cerebrospinal-fluid SOD1 and plasma neurofilament light. The 28-week randomized trial did not show a statistically significant benefit on the prespecified primary ALSFRS-R endpoint. Exploratory extension analyses favored earlier treatment. Accelerated approval was based on neurofilament reduction as a surrogate endpoint; confirmatory clinical benefit remains to be established	[93,100,104,105]
		C9orf72 ASOs	Mainly preclinical studies (cellular and animal models)	Reduces toxic repeat RNA accumulation and dipeptide repeat protein (DPR) production, thereby decreasing RNA-mediated and protein-mediated neurotoxicity	[93,100]
		SOD1 ASOs	In vitro cellular studies and ALS animal models	Reduces SOD1 expression, improves motor neuron survival, and alleviates SOD1-mediated neurotoxicity	[93,100]
		miRNA mimics	Mainly in vitro and animal models	Restores abnormal miRNA regulation, regulates motor neuron function, neuromuscular junction maintenance, inflammation, and neuronal survival pathways	[100]
AD/PD/ALS (emerging strategy)	Direct epitranscriptomic therapy (m6A modulation)	METTL3 inhibition	Preclinical models and early clinical trial in cancer (not neurodegenerative diseases)	Modulates m6A deposition by inhibiting METTL3; may regulate abnormal RNA metabolism and neuronal stress responses	[107,108,109]
		FTO inhibition	In vitro and animal studies (mainly cancer/inflammation models)	Increases m6A levels by blocking FTO-mediated demethylation; may restore RNA methylation homeostasis and neuronal function	[106,107,110,111]
		ALKBH5 inhibition	Mainly in vitro studies	Enhances m6A modification by suppressing ALKBH5 activity; potential application in correcting RNA methylation imbalance	[106,107]

## Data Availability

No new data were created or analyzed in this study. Data sharing is not applicable to this article.

## References

[1] GBD 2021 Nervous System Disorders Collaborators (2024). Global, regional, and national burden of disorders affecting the nervous system, 1990–2021: A systematic analysis for the Global Burden of Disease Study 2021. Lancet Neurol..

[2] Peggion C., Calì T., Brini M. (2024). Mitochondria dysfunction and neuroinflammation in neurodegeneration: Who comes first?. Antioxidants.

[3] Hardy J., Escott-Price V. (2025). The genetics of neurodegenerative diseases is the genetics of age-related damage clearance failure. Mol. Psychiatry.

[4] Valdes P., Caldwell A.B., Liu Q., Fitzgerald M.Q., Ramachandran S., Karch C.M., Galasko D.R., Yuan S.H., Wagner S.L., Subramaniam S. (2025). Integrative multiomics reveals common endotypes across PSEN1, PSEN2, and APP mutations in familial Alzheimer’s disease. Alzheimers Res. Ther..

[5] Krüger C., Lim S.Y., Buhrmann A., Fahrig F.L., Gabbert C., Bahr N., Madoev H., Marras C., Klein C., Lohmann K. (2025). Updated MDSGene review on the clinical and genetic spectrum of LRRK2 variants in Parkinson’s disease. npj Park. Dis..

[6] Nijs M., Van Damme P. (2024). The genetics of amyotrophic lateral sclerosis. Curr. Opin. Neurol..

[7] Jonson C., Levine K.S., Lake J., Hertslet L., Jones L., Patel D., Kim J., Bandres-Ciga S., Terry N., Mata I.F. (2024). Assessing the lack of diversity in genetics research across neurodegenerative diseases: A systematic review of the GWAS Catalog and literature. Alzheimers Dement..

[8] Zhang Y., Mao Y., Fu Q., Zhang X., Zhang D., Yue Y., Yang C. (2024). Scoping review of epigenetics on neurodegenerative diseases: Research frontiers and publication status. Front. Neurosci..

[9] Martinez-Feduchi P., Jin P., Yao B. (2024). Epigenetic modifications of DNA and RNA in Alzheimer’s disease. Front. Mol. Neurosci..

[10] Behl T., Kyada A., Roopashree R., Nathiya D., Arya R., Kumar M.R., Khalid M., Gulati M., Sachdeva M., Fareed M. (2024). Epigenetic biomarkers in Alzheimer’s disease: Diagnostic and prognostic relevance. Ageing Res. Rev..

[11] Olawade D.B., Rashad I., Egbon E., Teke J., Ovsepian S.V., Boussios S. (2025). Reversing epigenetic dysregulation in neurodegenerative diseases: Mechanistic and therapeutic considerations. Int. J. Mol. Sci..

[12] Sommerer Y., Dobricic V., Schilling M., Ohlei O., Sabet S.S., Wesse T., Fuß J., Franzenburg S., Franke A., Parkkinen L. (2023). Entorhinal cortex epigenome-wide association study highlights four novel loci showing differential methylation in Alzheimer’s disease. Alzheimers Res. Ther..

[13] Wang E., Wang M., Guo L., Fullard J.F., Micallef C., Bendl J., Song W., Ming C., Huang Y., Li Y. (2023). Genome-wide methylomic regulation of multiscale gene networks in Alzheimer’s disease. Alzheimer’s Dement..

[14] Zhao J., Gu T., Gao C., Miao G., Palma-Gudiel H., Yu L., Yang J., Wang Y., Li Y., Lim J. (2025). Brain 5-hydroxymethylcytosine alterations are associated with Alzheimer’s disease neuropathology. Nat. Commun..

[15] Zhang Z., Capuano A.W., Beadell A., Tasaki S., Cui X., Mehta R.I., Bennett D.A., He C., Zhang W., Arvanitakis Z. (2025). Brain-derived 5-hydroxymethylcytosine epigenetic scores are related to Alzheimer’s disease pathology and cognitive decline. Acta Neuropathol..

[16] Matatall K.A., Wathan T.K., Nguyen M., Chen H., McDonald A., Qi G., Belk J.A., Florez M.A., Le D.T., Olarinde T. (2025). TET2-mutant myeloid cells mitigate Alzheimer’s disease progression via CNS infiltration and enhanced phagocytosis in mice. Cell Stem Cell.

[17] Bonham L.W., Sirkis D.W., Pang A.P.S., Sugrue L.P., Santamaría-García H., Ibáñez A.M., Miller B.L., Yokoyama J.S., Corley M.J. (2025). DNA methylation age from peripheral blood predicts progression to Alzheimer’s disease, white matter disease burden, and cortical atrophy. npj Dement..

[18] Ramamurthy E., Welch G., Cheng J., Yuan Y., Gunsalus L., Bennett D.A., Tsai L.-H., Pfenning A.R. (2023). Cell type-specific histone acetylation profiling of Alzheimer’s disease subjects and integration with genetics. Front. Mol. Neurosci..

[19] Xu D.C., Sas-Nowosielska H., Donahue G., Huang H., Pourshafie N., Good C.R., Berger S.L. (2024). Histone acetylation in an Alzheimer’s disease cell model promotes homeostatic amyloid-reducing pathways. Acta Neuropathol. Commun..

[20] Pan R.Y., He L., Zhang J., Liu X., Liao Y., Gao J., Liao Y., Yan Y., Li Q., Zhou X. (2022). Positive feedback regulation of microglial glucose metabolism by histone H4 lysine 12 lactylation in Alzheimer’s disease. Cell Metab..

[21] Yang G., Ren Y., Zhong P., Patel P.J., Chen X.Q., Wan L., Lee Y.-H., Saleem K., Feng J., Yan Z. (2026). Histone demethylase PHF2 regulates inflammatory genes in Alzheimer’s disease. Mol. Psychiatry.

[22] Qin Y., Yang P., He W., Li D., Zeng L., Li J., Zhou T., Peng J., Cao L., Huang W. (2024). Novel histone post-translational modifications in Alzheimer’s disease: Current advances and implications. Clin. Epigenetics.

[23] Walgrave H., Balusu S., Snoeck S., Vanden Eynden E., Craessaerts K., Thrupp N., Wolfs L., Horré K., Fourne Y., Ronisz A. (2021). Restoring miR-132 expression rescues adult hippocampal neurogenesis and memory deficits in Alzheimer’s disease. Cell Stem Cell.

[24] Walgrave H., Penning A., Tosoni G., Snoeck S., Davie K., Davis E., Wolfs L., Sierksma A., Mars M., Bu T. (2023). microRNA-132 regulates gene expression programs involved in microglial homeostasis. iScience.

[25] Hu B., Shi Y., Xiong F., Chen Y.T., Zhu X., Carrillo E., Wen X., Drolet N., Rajpurohit C.S., Xu X. (2025). Rewired m6A of promoter antisense RNAs in Alzheimer’s disease regulates neuronal genes in 3D nucleome. Nat. Commun..

[26] Renganathan A., Minaya M.A., Broder M., Alfradique-Dunham I., Miller R.L., Dhavale D.D., Moritz M., Bhagat R., Marsh J., Verbeck A. (2025). A novel lncRNA FAM151B-DT regulates degradation of aggregation prone proteins. Mol. Psychiatry.

[27] Wang F., Li Y., Shen H., Martinez-Feduchi P., Ji X., Teng P., Krishnakumar S., Hu J., Chen L., Feng Y. (2024). Identification of pathological pathways centered on circRNAs dysregulation in association with irreversible progression of Alzheimer’s disease. Genome Med..

[28] Wu D.P., Wei Y.S., Hou L.X., Du Y.X., Yan Q.Q., Liu L.L., Zhao Y.-D., Yan R.-Y., Yu C., Zhong Z.-G. (2025). Circular RNA APP contributes to Alzheimer’s disease pathogenesis by modulating microglial polarization via miR-1906/CLIC1 axis. Alzheimers Res. Ther..

[29] Zhao F., Xu Y., Gao S., Qin L., Austria Q., Siedlak S.L., Pajdzik K., Dai Q., He C., Wang W. (2021). METTL3-dependent RNA m6A dysregulation contributes to neurodegeneration in Alzheimer’s disease through aberrant cell cycle events. Mol. Neurodegener..

[30] Wang X., Xie J., Tan L., Lu Y., Shen N., Li J., Hu H., Li H., Li X., Cheng L. (2023). N6-methyladenosine-modified circRIMS2 mediates synaptic and memory impairments by activating GluN2B ubiquitination in Alzheimer’s disease. Transl. Neurodegener..

[31] Yin H., Ju Z., Zheng M., Zhang X., Zuo W., Wang Y., Ding X., Zhang X., Peng Y., Li J. (2023). Loss of the m6A methyltransferase METTL3 in monocyte-derived macrophages ameliorates Alzheimer’s disease pathology in mice. PLoS Biol..

[32] Qiao Y., Mei Y., Xia M., Luo D., Gao L. (2024). The role of m6A modification in the risk prediction and Notch1 pathway of Alzheimer’s disease. iScience.

[33] Shafik A.M., Zhou H., Lim J., Dickinson B., Jin P. (2022). Dysregulated mitochondrial and cytosolic tRNA m1A methylation in Alzheimer’s disease. Hum. Mol. Genet..

[34] PerezGrovas-Saltijeral A., Rajkumar A.P., Knight H.M. (2023). Differential expression of m5C RNA methyltransferase genes NSUN6 and NSUN7 in Alzheimer’s disease and traumatic brain injury. Mol. Neurobiol..

[35] Smith A.R., Richards D.M., Lunnon K., Schapira A.H.V., Migdalska-Richards A. (2023). DNA methylation of α-synuclein intron 1 is significantly decreased in the frontal cortex of Parkinson’s individuals with GBA1 mutations. Int. J. Mol. Sci..

[36] Kochmanski J., Kuhn N.C., Bernstein A.I. (2022). Parkinson’s disease-associated, sex-specific changes in DNA methylation at PARK7 (DJ-1), SLC17A6 (VGLUT2), PTPRN2 (IA-2β), and NR4A2 (NURR1) in cortical neurons. npj Park. Dis..

[37] Min S., Xu Q., Qin L., Li Y., Li Z., Chen C., Wu H., Han J., Zhu X., Jin P. (2022). Altered hydroxymethylome in the substantia nigra of Parkinson’s disease. Hum. Mol. Genet..

[38] Choza J.I., Virani M., Kuhn N.C., Adams M., Kochmanski J., Bernstein A.I. (2025). Parkinson’s disease-associated alterations in DNA methylation and hydroxymethylation in human brain. npj Park. Dis..

[39] Andersen M.S., Leikfoss I.S., Brorson I.S., Cappelletti C., Bettencourt C., Toft M., Pihlstrøm L. (2023). Epigenome-wide association study of peripheral immune cell populations in Parkinson’s disease. npj Park. Dis..

[40] Gonzalez-Latapi P., Bustos B., Dong S., Lubbe S., Simuni T., Krainc D. (2024). Alterations in blood methylome as potential epigenetic biomarker in sporadic Parkinson’s disease. Ann. Neurol..

[41] Toker L., Tran G.T., Sundaresan J., Tysnes O.B., Alves G., Haugarvoll K., Nido G.S., Dölle C., Tzoulis C. (2021). Genome-wide histone acetylation analysis reveals altered transcriptional regulation in the Parkinson’s disease brain. Mol. Neurodegener..

[42] Huang M., Lou D., Charli A., Kong D., Jin H., Zenitsky G., Anantharam V., Kanthasamy A., Wang Z., Kanthasamy A.G. (2021). Mitochondrial dysfunction-induced H3K27 hyperacetylation perturbs enhancers in Parkinson’s disease. JCI Insight.

[43] Zhu J., Xu F., Lai H., Yuan H., Li X.Y., Hu J., Li W., Liu L., Wang C. (2023). ACO2 deficiency increases vulnerability to Parkinson’s disease via dysregulating mitochondrial function and histone acetylation-mediated transcription of autophagy genes. Commun. Biol..

[44] Guhathakurta S., Kim J., Adams L., Basu S., Song M.K., Adler E., Je G., Fiadeiro M.B., Kim Y. (2021). Targeted attenuation of elevated histone marks at SNCA alleviates α-synuclein in Parkinson’s disease. EMBO Mol. Med..

[45] Zhang Z., Wang R., Zhou H., Wu D., Cao Y., Zhang C., Sun H., Mu C., Hao Z., Ren H. (2024). Inhibition of EHMT1/2 rescues synaptic damage and motor impairment in a PD mouse model. Cell. Mol. Life Sci..

[46] Qin Q., Wang D., Qu Y., Li J., An K., Mao Z., Li J., Xiong Y., Min Z., Xue Z. (2025). Enhanced glycolysis-derived lactate promotes microglial activation in Parkinson’s disease via histone lactylation. npj Park. Dis..

[47] Zhang J., Zhao M., Yan R., Liu J., Maddila S., Junn E., Mouradian M.M. (2021). MicroRNA-7 protects against neurodegeneration induced by α-synuclein preformed fibrils in the mouse brain. Neurotherapeutics.

[48] Wang D., Gao H., Qin Q., Li J., Zhao J., Qu Y., Li J., Xiong Y., Min Z., Mao Z. (2024). MicroRNA-218-5p–Ddx41 axis restrains microglia-mediated neuroinflammation through downregulating type I interferon response in a mouse model of Parkinson’s disease. J. Transl. Med..

[49] Dong H., Yan J., Huang P., Wang X., Zhang R., Zhang C., Wang W., Qian W., Zhou J., Zhao Y. (2024). miR-214-3p promotes the pathogenesis of Parkinson’s disease by inhibiting autophagy. Biomed. Pharmacother..

[50] Duan R., Shi L., Deng Y., Wu J., Wang S., Peng Q., Li Z., Xu Z., Wang F., Xue X. (2024). AVE0991 ameliorates dopaminergic neuronal damage in Parkinson’s disease through HOTAIRM1/miR-223-3p/α-synuclein axis. Sci. Rep..

[51] Dong X., Bai Y., Liao Z., Gritsch D., Liu X., Wang T., Borges-Monroy R., Ehrlich A., Serrano G.E., Feany M.B. (2023). Circular RNAs in the human brain are tailored to neuron identity and neuropsychiatric disease. Nat. Commun..

[52] Liu Q., Li Q., Zhang R., Wang H., Li Y., Liu Z., Xie W., Geng D., Wang L. (2022). circ-Pank1 promotes dopaminergic neuron neurodegeneration through modulating miR-7a-5p/α-syn pathway in Parkinson’s disease. Cell Death Dis..

[53] Cheng Q., Wang J., Li M., Fang J., Ding H., Meng J., Zhang J., Fang X., Liu H., Ma C. (2022). CircSV2b participates in oxidative stress regulation through miR-5107-5p–Foxk1–Akt1 axis in Parkinson’s disease. Redox Biol..

[54] Martinez De La Cruz B., Gell C., Markus R., Macdonald I., Fray R., Knight H.M. (2023). m6A mRNA methylation in human brain is disrupted in Lewy body disorders. Neuropathol. Appl. Neurobiol..

[55] He H., Zhang Q., Liao J., Lei J., Luo M., Huang J., Chen M., Shen Y., Wang J., Xu P. (2023). METTL14 is decreased and regulates m6A modification of α-synuclein in Parkinson’s disease. J. Neurochem..

[56] Liu S., Ren Q., Mo G., Li Z., Huang H., Zhou Y., Miao Z., Cao X., Wu B., Xiao Z. (2026). m6A deficiency induces dopaminergic neurodegeneration and progressive parkinsonism through a pathogenic loop with mitochondria. J. Clin. Investig..

[57] Teng Y., Liu Z., Wei F., Tang Q., Li M., Chen X., Yi J., He S., Xu J., Hang Y. (2026). Loss of METTL14 in dopaminergic neurons disrupts ER homeostasis via m6A-dependent regulation of Atp2a3 mRNA: Implications for Parkinson’s disease. npj Park. Dis..

[58] Geng Y., Long X., Zhang Y., Wang Y., You G., Guo W., Zhuang G., Zhang Y., Cheng X., Yuan Z. (2023). FTO-targeted siRNA delivery by MSC-derived exosomes synergistically alleviates dopaminergic neuronal death in Parkinson’s disease via m6A-dependent regulation of ATM mRNA. J. Transl. Med..

[59] Li Z., Chen X., Xiang W., Tang T., Gan L. (2025). m6A demethylase FTO-mediated upregulation of BAP1 induces neuronal ferroptosis via the p53/SLC7A11 axis in the MPP+/MPTP-induced Parkinson’s disease model. ACS Chem. Neurosci..

[60] Miller C., Ealy A., Gregory A., Janarthanam C., Albers W., Richardson G., Jin H., Zenitsky G., Anantharam V., Kanthasamy A. (2025). Pathological α-synuclein dysregulates epitranscriptomic writer METTL3 to drive neuroinflammation in microglia. Cell Rep..

[61] Hop P.J., Zwamborn R.A.J., Hannon E., Shireby G.L., Nabais M.F., Walker E.M., van Rheenen W., van Vugt J.J., Dekker A.M., Westeneng H.-J. (2022). Genome-wide study of DNA methylation shows alterations in metabolic, inflammatory, and cholesterol pathways in ALS. Sci. Transl. Med..

[62] Yang T., Li C., Wei Q., Pang D., Cheng Y., Huang J., Lin J., Xiao Y., Jiang Q., Wang S. (2024). Genome-wide DNA methylation analysis related to ALS patient progression and survival. J. Neurol..

[63] Martin L.J., Adams D.A., Niedzwiecki M.V., Wong M. (2022). Aberrant DNA and RNA methylation occur in spinal cord and skeletal muscle of human SOD1 mouse models of ALS and in human ALS: Targeting DNA methylation is therapeutic. Cells.

[64] Stoccoro A., Smith A.R., Mosca L., Marocchi A., Gerardi F., Lunetta C., Cereda C., Gagliardi S., Lunnon K., Migliore L. (2020). Reduced mitochondrial D-loop methylation levels in sporadic amyotrophic lateral sclerosis. Clin. Epigenetics.

[65] Udine E., Finch N.A., DeJesus-Hernandez M., Jackson J.L., Baker M.C., Saravanaperumal S.A., Wieben E., Ebbert M.T., Shah J., Petrucelli L. (2024). Targeted long-read sequencing to quantify methylation of the C9orf72 repeat expansion. Mol. Neurodegener..

[66] Zhang M., McKeever P.M., Xi Z., Moreno D., Sato C., Bergsma T., McGoldrick P., Keith J., Robertson J., Zinman L. (2020). DNA methylation age acceleration is associated with ALS age of onset and survival. Acta Neuropathol..

[67] Zhao Y., Li X., Wang K., Iyer G., Sakowski S.A., Zhao L., Teener S., Bakulski K.M., Dou J.F., Traynor B.J. (2024). Epigenetic age acceleration is associated with occupational exposures, sex, and survival in amyotrophic lateral sclerosis. EBioMedicine.

[68] Rossaert E., Pollari E., Jaspers T., Van Helleputte L., Jarpe M., Van Damme P., De Bock K., Moisse M., Van Den Bosch L. (2019). Restoration of histone acetylation ameliorates disease and metabolic abnormalities in a FUS mouse model. Acta Neuropathol. Commun..

[69] Hou Y., Li Y., Xiang J.F., Tilahun K., Jiang J., Corces V.G., Yao B. (2024). TDP-43 chronic deficiency leads to dysregulation of transposable elements and gene expression by affecting R-loop and 5hmC crosstalk. Cell Rep..

[70] Li J., Jaiswal M.K., Chien J.F., Kozlenkov A., Jung J., Zhou P., Gardashli M., Pregent L.J., Engelberg-Cook E., Dickson D.W. (2023). Divergent single-cell transcriptome and epigenome alterations in ALS and FTD patients with C9orf72 mutation. Nat. Commun..

[71] Belzil V.V., Bauer P.O., Prudencio M., Gendron T.F., Stetler C.T., Yan I.K., Pregent L., Daughrity L., Baker M.C., Rademakers R. (2013). Reduced C9orf72 gene expression in c9FTD/ALS is caused by histone trimethylation, an epigenetic event detectable in blood. Acta Neuropathol..

[72] Marzullo M., Romano G., Pellacani C., Riccardi F., Ciapponi L., Feiguin F. (2023). Su(var)3-9 mediates age-dependent increase in H3K9 methylation on TDP-43 promoter triggering neurodegeneration. Cell Death Discov..

[73] Fisher R.M.A., Torrente M.P. (2024). Histone post-translational modification and heterochromatin alterations in neurodegeneration: Revealing novel disease pathways and potential therapeutics. Front. Mol. Neurosci..

[74] Amin N.D., Senturk G., Costaguta G., Driscoll S., O’Leary B., Bonanomi D., Pfaff S.L. (2021). A hidden threshold in motor neuron gene networks revealed by modulation of miR-218 dose. Neuron.

[75] Ionescu A., Ankol L., Ganapathy Subramaniam A., Altman T., Magen I., Cohen Y., Danino Y., Gradus-Pery T., Niv Y., Bar Avi O. (2025). Muscle-derived miR-126 regulates TDP-43 axonal local synthesis and NMJ integrity in ALS models. Nat. Neurosci..

[76] Li C., Wei Q., Hou Y., Lin J., Ou R., Zhang L., Jiang Q., Xiao Y., Liu K., Chen X. (2023). Genome-wide analyses identify NEAT1 as genetic modifier of age at onset of amyotrophic lateral sclerosis. Mol. Neurodegener..

[77] Kawakami Y., Iguchi Y., Li J., Amakusa Y., Yoshimura T., Chikuchi R., Yokoi S., Iida M., Riku Y., Iwasaki Y. (2025). Downregulation of NEAT1 due to loss of TDP-43 function exacerbates motor neuron degeneration in amyotrophic lateral sclerosis. Brain Commun..

[78] Hodgson R.E., Huang W.P., Lang R., Kumar V., An H., Stender E.G.P., Chalakova Z.P., Driver M.D., Avila A.S., Ellis B.C.S. (2026). Paraspeckle condensation is controlled via TDP-43 polymerization and linked to neuroprotection. Nat. Cell Biol..

[79] Wang S., Latallo M.J., Zhang Z., Huang B., Bobrovnikov D.G., Dong D., Livingston N.M., Tjoeng W., Hayes L.R., Rothstein J.D. (2021). Nuclear export and translation of circular repeat-containing intronic RNA in C9ORF72-ALS/FTD. Nat. Commun..

[80] Moreno-García L., Moreno-Martínez L., de la Torre M., Macías-Redondo S., García-Redondo A., Osta R., Toivonen J.M., Calvo A.C. (2025). Circular RNA Expression in ALS Is Progressively Deregulated and Tissue-Dependent. BMC Genom..

[81] Li Y., Dou X., Liu J., Xiao Y., Zhang Z., Hayes L., Wu R., Fu X., Ye Y., Yang B. (2023). Globally reduced N6-methyladenosine (m6A) in C9ORF72-ALS/FTD dysregulates RNA metabolism and contributes to neurodegeneration. Nat. Neurosci..

[82] McMillan M., Gomez N., Hsieh C., Bekier M., Li X., Miguez R., Tank E.M., Barmada S.J. (2023). RNA methylation influences TDP43 binding and disease pathogenesis in models of amyotrophic lateral sclerosis and frontotemporal dementia. Mol. Cell.

[83] Di Timoteo G., Giuliani A., Setti A., Biagi M.C., Lisi M., Santini T., Grandioso A., Mariani D., Castagnetti F., Perego E. (2024). m6A reduction relieves FUS-associated ALS granules. Nat. Commun..

[84] Dermentzaki G., Furlan M., Tanaka I., Leonardi T., Rinchetti P., Passos P.M.S., Bastos A., Ayala Y.M., Hanna J.H., Przedborski S. (2024). Depletion of Mettl3 in cholinergic neurons causes adult-onset neuromuscular degeneration. Cell Rep..

[85] Yen Y.P., Lung T.H., Liau E.S., Wu C.C., Huang G.L., Hsu F.Y., Chang M., Yang Z.-D., Huang C.-Y., Zheng Z. (2025). The motor neuron m6A repertoire governs neuronal homeostasis and FTO inhibition mitigates ALS symptom manifestation. Nat. Commun..

[86] Parra-Torres M., Dissanayake K., Gray J.A., Langlands A.J., Kucuk R., Gierlinski M., Troakes C., King A., McGurk L. (2025). Aberrant NSUN1 activity connects m5C-RNA modification to TDP-43 neurotoxicity in ALS/FTD. Life Sci. Alliance.

[87] Berson E., Sreenivas A., Phongpreecha T., Perna A., Grandi F.C., Xue L., Ravindra N.G., Payrovnaziri N., Mataraso S., Kim Y. (2023). Whole genome deconvolution unveils Alzheimer’s resilient epigenetic signature. Nat. Commun..

[88] Xiong X., James B.T., Boix C.A., Park Y.P., Galani K., Victor M.B., Sun N., Hou L., Ho L.-L., Mantero J. (2023). Epigenomic dissection of Alzheimer’s disease pinpoints causal variants and reveals epigenome erosion. Cell.

[89] Shwab E.K., Gingerich D.C., Man Z., Gamache J., Garrett M.E., Crawford G.E., Ashley-Koch A.E., Serrano G.E., Beach T.G., Lutz M.W. (2024). Single-nucleus multi-omics of Parkinson’s disease reveals a glutamatergic neuronal subtype susceptible to gene dysregulation via alteration of transcriptional networks. Acta Neuropathol. Commun..

[90] Smith A.R., Smith R.G., Macdonald R., Marzi S.J., Burrage J., Troakes C., Al-Sarraj S., Mill J., Lunnon K. (2021). The histone modification H3K4me3 is altered at the ANK1 locus in Alzheimer’s disease brain. Future Sci. OA.

[91] Han S.W., Pyun J.M., Bice P.J., Bennett D.A., Saykin A.J., Kim S.Y., Park Y.H., Nho K. (2024). miR-129-5p as a biomarker for pathology and cognitive decline in Alzheimer’s disease. Alzheimers Res. Ther..

[92] Dai W., Qiao X., Fang Y., Guo R., Bai P., Liu S., Li T., Jiang Y., Wei S., Na Z. (2024). Epigenetics-targeted drugs: Current paradigms and future challenges. Signal Transduct. Target. Ther..

[93] Ertural B., Çiçek B.N., Kurnaz I.A. (2025). RNA therapeutics: Focus on antisense oligonucleotides in the nervous system. Biomol. Ther..

[94] Kaur G., Rathod S.S.S., Ghoneim M.M., Alshehri S., Ahmad J., Mishra A., Alhakamy N.A. (2022). DNA methylation: A promising approach in management of Alzheimer’s disease and other neurodegenerative disorders. Biology.

[95] Wang Y., Wang X., Li R., Yang Z.F., Wang Y.Z., Gong X.L., Wang X. (2013). A DNA methyltransferase inhibitor, 5-aza-2′-deoxycytidine, exacerbates neurotoxicity and upregulates Parkinson’s disease-related genes in dopaminergic neurons. CNS Neurosci. Ther..

[96] Diniz L.P., Pinheiro P.S.M., Franco L.S., Gomes F.C.A. (2025). HDACs in the brain: From chromatin remodeling to neurodegenerative disease. Cells.

[97] Pinheiro P.S.M., Diniz L.P., Franco L.S., Siqueira M., Gomes F.C.A. (2025). Histone deacetylases in neurodegenerative diseases and their potential role as therapeutic targets: Shedding light on astrocytes. Pharmaceuticals.

[98] Francelle L., Outeiro T.F., Rappold G.A. (2020). Inhibition of HDAC6 activity protects dopaminergic neurons from alpha-synuclein toxicity. Sci. Rep..

[99] Piepers S., Veldink J.H., de Jong S.W., van der Tweel I., van der Pol W.L., Uijtendaal E.V., Schelhaas H.J., Scheffer H., De Visser M., De Jong J.M.B.V. (2009). Randomized sequential trial of valproic acid in amyotrophic lateral sclerosis. Ann. Neurol..

[100] Saadh M.J., Qahtan S.A., Albadr R.J., Sanghvi G., Roopashree R., Kashyap A., Sabarivani A., Rizaev J., Taher W.M., Alwan M. (2025). Non-coding RNAs as key players in neurodegeneration and brain tumors: Insights into therapeutic strategies. Iran. J. Basic. Med. Sci..

[101] Mummery C.J., Börjesson-Hanson A., Blackburn D.J., Vijverberg E.G.B., De Deyn P.P., Ducharme S., Jonsson M., Schneider A., Rinne J.O., Ludolph A.C. (2023). Tau-targeting antisense oligonucleotide MAPTRx in mild Alzheimer’s disease: A phase 1b, randomized, placebo-controlled trial. Nat. Med..

[102] Cole T.A., Zhao H., Collier T.J., Sandoval I., Sortwell C.E., Steece-Collier K., Daley B.F., Booms A., Lipton J., Welch M. (2021). α-Synuclein antisense oligonucleotides as a disease-modifying therapy for Parkinson’s disease. JCI Insight.

[103] Sano T., Nagata T., Ebihara S., Yoshida-Tanaka K., Nakamura A., Sasaki A., Shimozawa A., Mochizuki H., Uchihara T., Hasegawa M. (2024). Effects of local reduction of endogenous α-synuclein using antisense oligonucleotides on the fibril-induced propagation of pathology through the neural network in wild-type mice. Acta Neuropathol. Commun..

[104] Miller T.M., Cudkowicz M.E., Genge A., Shaw P.J., Sobue G., Bucelli R.C., Chiò A., Van Damme P., Ludolph A.C., Glass J.D. (2022). Trial of antisense oligonucleotide tofersen for SOD1 ALS. N. Engl. J. Med..

[105] Blair H.A. (2023). Tofersen: First approval. Drugs.

[106] Gao Z., Zha X., Li M., Xia X., Wang S. (2024). Insights into the m6A demethylases FTO and ALKBH5: Structural, biological function, and inhibitor development. Cell Biosci..

[107] Zhang J., Ma W., Liu R., Li X., Yuan Z., Cheng J. (2026). Neuromodulatory role and therapeutic potential of N6-methyladenosine RNA methylation in neurodegenerative diseases. Neural Regen. Res..

[108] Yankova E., Blackaby W., Albertella M., Rak J., De Braekeleer E., Tsagkogeorga G., Pilka E.S., Aspris D., Leggate D., Hendrick A.G. (2021). Small-molecule inhibition of METTL3 as a strategy against myeloid leukaemia. Nature.

[109] Moser J.C., Papadopoulos K.P., Rodon Ahnert J., Ofir-Rosenfeld Y., Holz J.B., STC15-22101 Study Team (2024). Phase 1 dose escalation and cohort expansion study evaluating safety, pharmacokinetics, pharmacodynamics and clinical activity of STC-15, a METTL3 inhibitor, in patients with advanced malignancies. J. Clin. Oncol..

[110] Huang Y., Su R., Sheng Y., Dong L., Dong Z., Xu H., Ni T., Zhang Z.S., Zhang T., Li C. (2019). Small-molecule targeting of oncogenic FTO demethylase in acute myeloid leukemia. Cancer Cell.

[111] Lian Z., Chen R., Xian M., Huang P., Xu J., Xiao X., Ning X., Zhao J., Xie J., Duan J. (2024). Targeted inhibition of m6A demethylase FTO by FB23 attenuates allergic inflammation in the airway epithelium. FASEB J..

[112] Yang H.M. (2025). Overcoming the blood–brain barrier: Advanced strategies in targeted drug delivery for neurodegenerative diseases. Pharmaceutics.

[113] Zhang X., Fu M., Wang Y., Wu T. (2025). Strategies for delivering drugs across the blood–brain barrier for the treatment of neurodegenerative diseases. Front. Drug Deliv..

[114] Qasim H., Khattab K., Abu Shugaer M., Varrassi G. (2025). Epigenetic dysregulation in neurodegenerative disease: Implications for neuropathology and therapy. Cureus.

[115] Wang J., Wang S., Li Q., Liu F., Wan Y., Liang H. (2024). Bibliometric and visual analysis of single-cell multiomics in neurodegenerative disease arrest studies. Front. Neurol..

[116] Ren F., Wei J., Chen Q., Hu M., Yu L., Mi J., Zhou X., Qin D., Wu J., Wu A. (2025). Artificial intelligence-driven multi-omics approaches in Alzheimer’s disease: Progress, challenges, and future directions. Acta Pharm. Sin. B.

[117] Marei H.E. (2025). Epigenetic editing in neurological and neuropsychiatric disorders: Pioneering next-gen therapeutics for precision gene control. Mol. Neurobiol..

